# Age-Associated Heterogeneity of Ty21a-Induced T Cell Responses to HLA-E Restricted *Salmonella* Typhi Antigen Presentation

**DOI:** 10.3389/fimmu.2019.00257

**Published:** 2019-03-04

**Authors:** Mark E. Rudolph, Monica A. McArthur, Laurence S. Magder, Robin S. Barnes, Wilbur H. Chen, Marcelo B. Sztein

**Affiliations:** ^1^Center for Vaccine Development and Global Health, University of Maryland School of Medicine, Baltimore, MD, United States; ^2^Molecular Microbiology and Immunology Department, University of Maryland Graduate Program in Life Sciences, Baltimore, MD, United States; ^3^Department of Pediatrics, University of Maryland School of Medicine, Baltimore, MD, United States; ^4^Epidemiology and Public Health, University of Maryland School of Medicine, Baltimore, MD, United States; ^5^Department of Medicine, University of Maryland School of Medicine, Baltimore, MD, United States

**Keywords:** T cell response, dimensionality reduction, Ty21a, multifunctionality, pediatric immunology, *Salmonella* typhi, HLA-E restricted responses, typhoid

## Abstract

Human-restricted *Salmonella enterica* serovar Typhi (*S*. Typhi) is the causative agent of typhoid fever—a life-threatening disease of great global health significance, particularly in the developing world. Ty21a is an oral live-attenuated vaccine that protects against the development of typhoid disease in part by inducing robust T cell responses, among which multifunctional CD8^+^ cytotoxic T lymphocytes (CTL) play an important role. Following Ty21a vaccination, a significant component of adult CTL have shown to be targeted to *S*. Typhi antigen presented by the conserved major histocompatibility complex (MHC) class Ib molecule, human leukocyte antigen-E (HLA-E). *S*. Typhi challenge studies have shown that baseline, multifunctional HLA-E responsive T cells are associated with protection from, and delayed onset of, typhoid disease. However, despite the overwhelming burden of typhoid fever in school-aged children, and due to limited availability of pediatric samples, incomplete information is available regarding these important HLA-E-restricted responses in children, even though studies have shown that younger children may be less likely to develop protective cell mediated immune (CMI) responses than adults following vaccination. To address this gap, we have studied this phenomenon in depth by using mass cytometry to analyze pediatric and adult T cell responses to HLA-E-restricted *S*. Typhi antigen presentation, before and after Ty21a vaccination. Herein, we show variable responses in all age strata following vaccination among T effector memory (T_EM_) and T effector memory CD45RA^+^ (T_EMRA_) cells based on conventional gating analysis. However, by utilizing the dimensionality reduction tool tSNE (t-distributed Stochastic Neighbor Embedding), we are able to identify diverse, highly multifunctional gut-homing- T_EM_ and T_EMRA_ clusters of cells which are more abundant in adult and older pediatric participants than in younger children. These findings highlight a potential age-associated maturation of otherwise conserved HLA-E restricted T cell responses. Such insights, coupled with the marked importance of multifunctional T cell responses to combat infection, may better inform future pediatric vaccination strategies against *S*. Typhi and other infectious diseases.

## Introduction

*Salmonella enterica* serovar Typhi (*S*. Typhi) is a human-restricted obligate-intracellular pathogen and is the causative agent of typhoid fever, which causes around 21-million illnesses and over 200,000 deaths per year (1–4). The pathogen is spread via contaminated food and water, and much of the disease burden lies on the developing world with significant morbidity associated among school-aged children (1, 3, 5–9). Challenge studies have shown that robust, multifunctional baseline CD8^+^ T cell responses to *S*. Typhi antigen presentation are associated with protection from, and/or delayed onset of typhoid disease (10, 11). Therefore, a successful attenuated typhoid vaccine should aim to induce these cell-mediated immunological (CMI) responses. Indeed, the oral live-attenuated Ty21a vaccine has been shown to induce CMI in approximately two-thirds of adult recipients (12–14).

Because typhoid fever is a disease that disproportionally impacts children in the developing world, successful vaccination strategies require protecting pediatric populations in endemic regions. Unfortunately, limited information has been reported addressing potential differences between pediatric and adult T cell responses to vaccination, especially regarding oral, live-attenuated vaccines. Previous studies in healthy volunteers have shown that the percentage of total T cells (CD3^+^) and CD8^+^ T cells in peripheral blood are stable throughout life (15–17). However, in general peripheral naïve T cell populations are higher in younger children, with memory T cell phenotypes increasing throughout life (15–20). Previous work from our group has shown that following mitogenic stimulation, CD8^+^ T cell activation—as defined by CD69 expression—increases throughout adolescence, plateauing by the age of 15-years-old (17). Further, we identified significantly lower mitogen-induced CD8^+^ T_EM_ multifunctional effector responses in younger children compared to adults (17). Interestingly, studies have suggested that younger children may be less likely than older children and adults to develop lasting, protective CMI following vaccination (21, 22), and field trial data of Ty21a showed lower efficacy in children aged 9–14 than those aged 15 or older (4, 23, 24).

HLA-E is a highly conserved non-classical class I MHC, capable of presenting the MHC leader peptide to CD94/NKG2 as a means of regulating natural killer cell function (25). In addition to this innate, inhibitory presentation of self-antigens, HLA-E can present a limited repertoire of peptides from a variety of viral and bacterial pathogens to CD8^+^ T cells, leading to activation of adaptive immune responses separate from those that require classical HLA restriction (26–29). Previous work from our laboratory identified seven HLA-E binding peptides, derived from the *S*. Typhi GroEL sequence, that elicit cytotoxic CD8^+^ T lymphocyte responses following Ty21a vaccination—and that this restricted response makes up ~30% of the total CD8+ T cell response (30). Further studies have found that Ty21a vaccination is capable of inducing multifunctional *S*. Typhi-reactive HLA-E restricted CD8^+^ T_EM_ and T_EMRA_ in adults for up to 2 years post-vaccination (31). Additionally, we have shown HLA-E restricted responders show an earlier post-Ty21a immunization peak than those responding to *S*. Typhi infected autologous B-lymphoblastoid cell line (B-LCL) (32). Finally, the presence of baseline *S*. Typhi-responsive HLA-E-restricted T cells is associated with delayed onset of disease in a low-dose human *S*. Typhi challenge model (10).

In this study we utilized an established *S*. Typhi-infected HLA-E-restricted antigen presentation model (10, 11, 30–32) to explore the variability of CD8^+^ T cell responses among pediatric and adult Ty21a vaccine recipients. To explore these responses in depth we used a comprehensive mass cytometry panel which enables us to characterize T cell memory, activation, and proliferation, as well as the expression of homing molecules and the production of multiple effector cytokines and chemokines. Additionally, we used a variety of dimensionality reduction techniques to characterize multifunctional responders, as well as to further explore distinct Ty21a-responsive populations within our participant cohorts. Herein we describe how Ty21a vaccination elicits age-associated differences among HLA-E restricted CD8^+^ T cell effector responses, multifunctionality, and homing potential, and discuss how these differences may influence future pediatric *S*. Typhi vaccination strategies.

## Materials and Methods

### Participants and Isolation of PBMC

PBMC were collected before Ty21a vaccination, and between 14 and 42 days following Ty21a vaccination, from 18 healthy pediatric [6–17 years of age at the time of enrollment, receiving Ty21a vaccination for medically indicated reasons (i.e., travel to endemic regions)] and 13 healthy adult (20–65 years of age at the time of enrollment) volunteers, as listed in Table 1, following recruitment from the Baltimore-Washington area and the University of Maryland at Baltimore campus. These studies were approved by the University of Maryland at Baltimore Institutional Review Board (IRB) and were carried out in accordance with the Declaration of Helsinki. Written and informed consent was obtained from all adult participants, as well as written informed consent from the parents of any participant under the age of 18 years old, and assent from the pediatric participants, prior to the conduct of any study procedures. PBMC were isolated immediately following blood collection by density gradient centrifugation and stored in liquid nitrogen following standard cryopreservation techniques (33, 34) until used in the assays. Where cell numbers permitted, B-lymphoblastoid cells generated from PBMC were HLA-E typed by DNA exon sequencing at the University of Oklahoma Health Sciences Center's Sequence-Based Typing facility directed by Dr. William Hildebrand.

**Table 1:** Participant Ty21a vaccinees listed by age (years), sex, race, and HLA-E haplotype.

**Age (Years)**	**Sex**	**Race**	**HLA-E Haplotype**
6	Male	African American	*01
13	Female	African American	Heterozygous
13	Female	African American	Heterozygous
13	Male	Caucasian	*03
14	Male	Hispanic	*03
14	Female	Caucasian	Heterozygous
14	Female	Caucasian	Heterozygous
15	Female	Caucasian	n/a
15	Female	Caucasian	*01
15	Female	Caucasian	n/a
16	Male	Caucasian	*01
16	Male	Caucasian	n/a
16	Male	Caucasian	*03
16	Male	African American	Heterozygous
16	Male	Caucasian	Heterozygous
17	Female	Caucasian	*01
17	Male	Caucasian	Heterozygous
17	Male	Caucasian/Hispanic	Heterozygous
20	Female	African American	*01
25	Female	African American	*01
27	Female	African American	*01
33	Female	Caucasian	Heterozygous
36	Male	Caucasian	*01
41	Female	Caucasian	*03
43	Male	African American	*01
43	Male	African American	Heterozygous
44	Female	African American	*01
51	Male	African American	Heterozygous
62	Male	n/a	*03
65	Male	n/a	Heterozygous
65	Male	n/a	*03

*n/a, not available*.

### Preparation of HLA-E-Restricted Target Cells

HLA class I-defective B cell line transfected with HLA-E fused to the HLA-A2 leader peptide (721.221.AEH), allowing for expression of the HLA-E^*^01:01 allele on the cell surface (35), was thawed in complete 1640 RPMI media (Gibco, Carlsbad, CA), supplemented with 100 U/mL penicillin (Sigma), 100 μg/mL streptomycin (Sigma, St. Louis, MO), 50 μg/mL gentamicin (Gibco), 2 mM L-glutamine (Gibco), 2.5 mM sodium pyruvate (Gibco), 10 mM HEPES buffer (Gibco), non-essential amino acids (Lonza, Basel, Switzerland), and 10% fetal bovine serum (Gemini Bioproducts, West Sacramento, CA) and allowed to expand. HLA-E restricted targets were infected in RPMI without antibiotics for 3 h at 37°C with wild-type *S*. Typhi strain ISP1820 (wt *S*. Typhi) at a multiplicity of infection (MOI) of 7:1 (3.5 × 10^7^ wt *S*. Typhi as determined by OD600: 5.0 × 10^6^ 721.221.AEH cells). Uninfected targets were used as controls. Following the 3 h incubation, all cells are washed twice and incubated at 37°C, 5% CO_2_ overnight in cRPMI supplemented with 0.3% gentamycin. Expression of wt *S*.Typhi antigens on live, infected targets (and not on uninfected targets) was confirmed by conventional flow cytometry with LIVE/DEAD™ yellow cell stain (ThermoFisher Scientific, Waltham, MA) and BacTrace® FITC-labeled anti-*Salmonella* CSA-1 antibody (SeraCare, Milford, MA). Cells were subsequently gamma-irradiated (6,000 rad) and re-suspended in fresh cRPMI before being used as targets in co-culture with effector cells.

### *In vitro* Stimulation

PBMC were thawed and rested overnight at 37°C, 5% CO_2_ in cRPMI. After the overnight rest, cells were washed and resuspended in cRPMI at a concentration of 1 × 10^6^ cells/500 μL in 5 mL in cell culture tubes. Irradiated target cells were incubated with PBMC at an effector-to-stimulator ratio of 5:1 in the presence of 3 μL/mL anti-CD107a monoclonal antibody (mAb) conjugated to 151Eu (Fluidigm, South San Francisco, CA) for 2 h at 37°C in 5% CO_2_. After the 2 h incubation, GolgiStop (containing monensin), and GolgiPlug (containing brefeldin A) from BD (San Jose, CA) were added at 0.5 μL/mL to all tubes and cultures were maintained at 37°C in 5% CO_2_ overnight.

### Surface and Intracellular Labeling and Mass Cytometry Analysis

Co-cultured PBMC were centrifuged and incubated with anti-CD45 (Fluidigm South San Francisco, CA) monoclonal antibodies (mAbs) for barcoding. Pediatric samples were stained with CD45-154Sm and adult samples were stained with CD45-156Gd for 30 min at 4°C. Cells were then washed once with flow cytometry buffer [1x PBS, Quality Biological, Gaithersburg, MD), 0.1% sodium azide (Sigma), 2% fetal bovine serum (Gemini Bioproducts)] and once with serum-free RPMI (Gibco) before being combined into their barcoded layout. Like-stimulated adult (CD45-156Gd) and pediatric (CD45-154Sm) PBMC were combined into a single tube for downstream staining. Mass cytometry staining was performed as described in the Materials and Methods section “Mass Cytometry Measurements” from McArthur et al. (36, 37) using the monoclonal antibodies listed in Table 2. Briefly, cells were labeled with metal-tagged antibodies against specific surface and intracellular targets, cisplatin as a viability marker, and iridium as a DNA intercalator for cell identification before preparation and running by the UMB flow cytometry and mass cytometry core in a CyTOF instrument (Fluidigm). Mass cytometry data were analyzed using WinList version 9.0.1 (Verity Software House, Topsham, ME) following debarcoding of the files, based on whether they were tagged with 154Sm (pediatric)- or 156Gd (adult)-labeled CD45, with Premium Cytobank (Cytobank, Inc, Santa Clara, CA). Net responses were calculated by subtracting T cell responses to uninfected HLA-E-restricted antigen presenting cells from responses to infected HLA-E-restricted targets. tSNE analysis was run in R using the Cytofkit package in biocLite (38). Gating of individual tSNE clusters was performed using WinList version 9.0.1.

**Table 2:** Mass cytometry panel showing antibody target, stable metal isotope (or other label), antibody clone, and a brief description of the target function.

**Target**	**Stable metal isotope**	**Clone**	**Description**
CD14	114 Cd (Qdot)	TüK4	Monocyte marker
CD19	114 Cd (Qdot)	SJ25-C1	B cell marker
IL-4	142 Nd	MP425D2	Tc/h2 effector cytokine
*CXCR5*	*Biotin*	*RF8B2*	Follicular homing chemokine receptor
Biotin	143 Nd	1D4-C5	
*α4β7*	*FITC*	*ACT-1*	Gut-homing integrin
FITC	144 Nd	FIT22	
CD8	146 Nd	RPA-T8	Cytotoxic T lymphocyte marker
IL-6	147 Sm	MQ213A5	Proinflammatory proliferation-associated cytokine
CCR4	149 Sm	L291H4	Chemokine homing to the skin
MIP-1β	150 Nd	D21-1351	NK and monocyte recruiting chemokine
CD107a	151 Eu	H4A3	Degranulation marker
TNFα	152 Sm	Mab11	Proinflammatory cytokine
CD62L	153 Eu	DREG-56	Lymphoid-tissue homing selectin
CD45	154 Sm	HI30	Pan-leukocyte barcoding marker
CD27	155 Gd	L128	TNF superfamily—costimulatory molecule
CD45	156 Gd	HI30	Pan-leukocyte barcoding marker
IL-2	158 Gd	MQ1-17H12	Induction of T cell differentiation and proliferation
CD69	162 Dy	FN50	Activation marker
CXCR3	163 Dy	G025H7	Chemokine homing to sites of inflammation
IL-17A	164 Dy	N49-653	Tc/h17 effector cytokine
IFNγ	165 Ho	B27	Proinflammatory cytokine
IL-10	166 Er	JES3-9D7	Anti-inflammatory cytokine
CD154 (CD40L)	168 Er	24-31	Co-stimulatory molecule; Tfh induction of B cell maturation
CD45RA	169 Tm	HI-100	T cell memory marker
CD3	170 Er	UCHT1	TCR co-receptor (T cell marker)
Granzyme B	171 Yb	GB11	Secreted cytotoxic effector molecule
IL-21	172 Yb	3A3-N2	Tfh effector cytokine (germinal center formation)
ICOS (CD278)	173 Yb	C398.4A	Tfh co-stimulatory marker (B cell help)
CD4	174 Yb	SK3	Helper T lymphocyte marker
PD-1*	175 Lu	EH12.2H7	T cell exhaustion/activation marker
CCR6	176 Yb	G034E3	Chemokine homing to mucosal surfaces
Cell I.D. (DNA)	191/193 Ir	n/a	DNA intercalator
Viability	194/195 Pt	n/a	Viability stain

Primary surface antibodies are filled with blue, secondary surface antibodies with green, and intracellular antibodies with orange (*anti-PD-1 is in both the surface and intracellular antibody mixes). Barcoding antibodies are shaded in yellow, and anti-CD107a, which is added during stimulation to best account for the active cycling between the surface and intracellular vesicles, is highlighted in purple.

### Statistical Analyses

All analyses were performed using GraphPad Prism version 7.0c. Unpaired *t*-tests, paired *t*-tests, *z*-scores, and/or chi squared tests were performed depending on the analysis as indicated. *P*-values of < 0.05 were considered significant.

## Results

### CD8^+^ T Cell Populations Before and After Ty21a Vaccination in Different Age Strata

**Figure 1:**
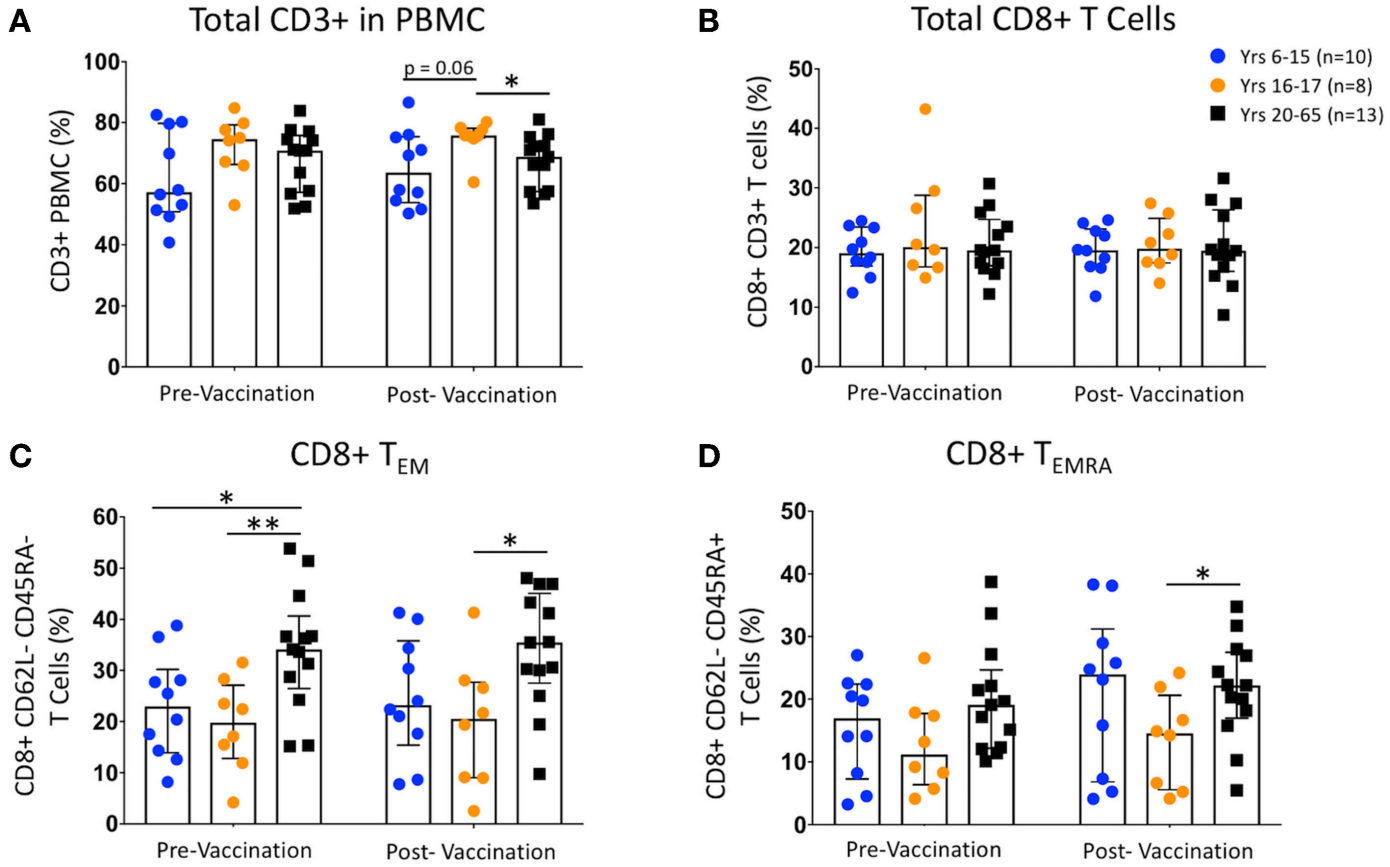
Change in CD8 T cell Populations Following Ty21a Vaccination. Scatter plots showing the percentages of **(A)** pre- and post-vaccination CD3+ T cells, **(B)** total CD8+ T cells, **(C)** CD8+ T effector memory (T_EM_; CD45RA- CD62L-), and **(D)** CD8+ T EMRA (T_EMRA_; CD45RA+ CD62L-) populations among 6–15 year-old pediatric (*n* = 10), 16–17 year-old pediatric (*n* = 8), and adult (*n* = 13) participants (media). Bars represent medians with whiskers indicating interquartile ranges. Statistics were analyzed by unpaired *t*-test (*p < 0.05; **p < 0.01).

Unstimulated PBMC from pre- and 14 to 42-days post-Ty21a vaccinated healthy pediatric and adult participants were labeled with metal-conjugated mAbs and analyzed on a mass cytometer. Percentages of total T cells (CD3^+^ CD14^−^ CD19^−^), total CD8^+^ T cells (CD4^−^ CD8^+^), CD8^+^ T_EM_ (CD62L^−^ CD45RA^−^), and T_EMRA_ (CD62L^−^ CD45RA^+^) cells showed no changes between vaccination states within either 6–15 year-old children (*n* = 10), 16–17 year-old children (*n* = 8), or adult (20–65 years old; *n* = 13) participants (Figures 1A–D). CD62L^−/lo^ populations are capable of defining effector memory populations similar to CCR7 expression, and the identification of T memory subsets using CD62L and CD45RA is well-established (39). While no differences were observed comparing pre- and post-vaccinated pediatric age groups, we note that, as has been previously reported by us and others (16, 17, 19, 20, 40, 41), the percentage of CD8^+^ T_EM_ are lower in children than in adults (Figure 1C). Further, while we see no differences within groups following vaccination, among unstimulated T cell populations divided by both sex and age (Supplemental Figures 1A–D), we observed that the greatest differences among the CD8^+^ T_EM_ are between pediatric males (ages 6–17; *n* = 10) and females (ages 11–17; *n* = 8), and adult males (ages 36–65; *n* = 7), regardless of vaccination status (Supplemental Figure 1C). Finally, the percentages of CD8^+^ T_EM_ and T_EMRA_ populations are not significantly different, nor does vaccination alter those percentages, among participants with different HLA-E haplotypes (Supplemental Figures 1E,F).

### Change in Activated CD8^+^ T Cell Populations Following Ty21a Vaccination

**Figure 2:**
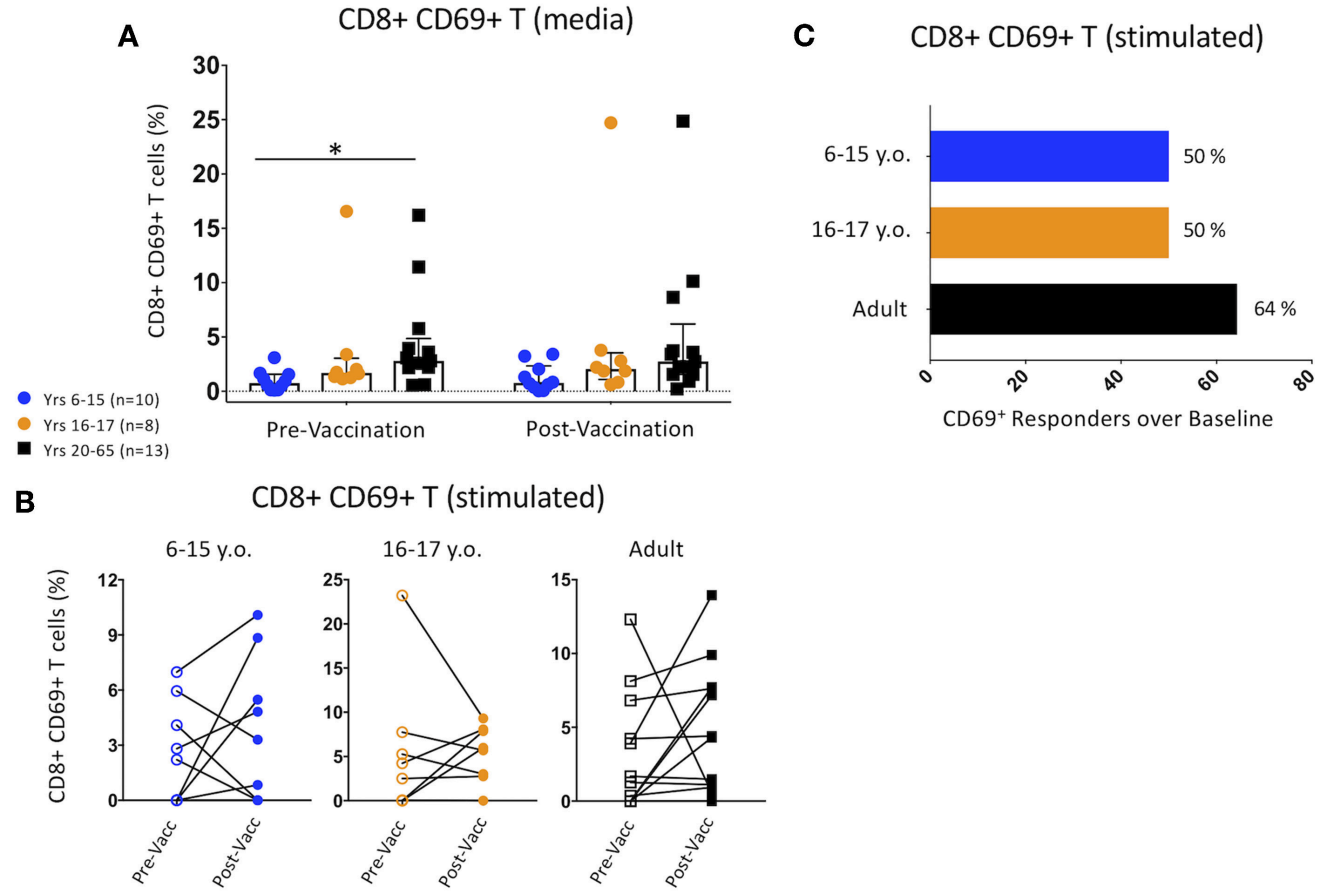
Changes in Activated CD8+ CD69+ T cell Populations Following Ty21a Vaccination. **(A)** Scatter plot showing the percentages of CD69+ CD8+ T cells among pre- and post-Ty21a unstimulated PBMC (media). Bars represent medians with whiskers indicating interquartile ranges. **(B)** Connected dot plots showing each participants' pre- and post-Ty21a CD8+ CD69+ expression levels following co-culture with *S*. Typhi-infected HLA-E restricted antigen presenting target cells. Data divided among 6–15 year-old pediatric (*n* = 10), 16–17 year-old pediatric (*n* = 8), and adult (*n* = 13) participants. **(C)** Bars representing the proportion of CD8+ CD69+ responders over baseline levels (cut-off of 0.1%) following co-culture with *S*. Typhi-infected HLA-E restricted antigen presenting target cells. Statistics were analyzed by unpaired *t*-test. (*p < 0.05).

CD69 expression was used to define activated CD8^+^ T cell populations among participants pre- and post-vaccination. Unstimulated adult PBMC pre-vaccination showed significantly higher CD69 expression than 6–15 year-old pediatric participants, and trended toward significance within the post-Ty21a time point (Figure 2A). These differences were maintained between pediatric females and adult males but were not seen among other gender and age divisions (Supplemental Figure 2A). There was no change in CD69 expression between pre- and post-vaccination in any of the age groups (Figure 2A).

We then explored whether Ty21a vaccination induced higher levels of *S*. Typhi-responsive activation among different age groups by co-culturing PBMC obtained pre- or post-vaccination with *S*. Typhi-infected HLA-E-restricted target cells. None of the age-associated changes among the pre- and post-vaccination activated CD8^+^ T cell percentages were significant (Figure 2B). However, when using a *z*-score between events collected in *S*. Typhi-infected vs. non-infected targets with a *p* < 0.05 and a cutoff of 0.1%, over baseline, we observed a trend for higher percentage of adult responders than were observed in either pediatric age group (Figure 2C).

No significant differences were observed between the percentages of CD69^+^ T cells at baseline or following vaccination based on sex or age (Supplemental Figure 2B). Further, the *z*-score between events collected in *S*. Typhi-infected vs. non-infected targets with a *p* < 0.05 and a cutoff of 0.1%, over baseline, did not show significant differences among gender- and age-parsed populations, despite some trends observed in the percentages of responders (particularly among pediatric females), likely due to insufficient numbers of participants in each group (Supplemental Figure 2C). Interestingly, while there were no differences in unstimulated CD8^+^ activation percentages among pre- and post-vaccination HLA-E haplotypes (Supplemental Figure 2D), we observed significant differences following stimulation with *S*. Typhi-infected targets between HLA-E^*^01:01 or heterozygous individuals and HLA-E^*^01:03 before immunization (Supplemental Figure 2E), However, when dividing the populations into HLA-E haplotypes, we observed that HLA-E^*^01:03 participants pre-vaccination show almost no baseline activation (as defined by CD69 expression), yet nearly all of these participants show >0.1% increases in the percentage of CD69^+^ CD8^+^ T cells over baseline following vaccination, suggesting a possible Ty21a-induced change in activation threshold among these HLA-E mismatched individuals (Supplemental Figures 2E,F).

### CD8^+^ T_EM_ Responses Following Ty21a Vaccination

**Figure 3:**
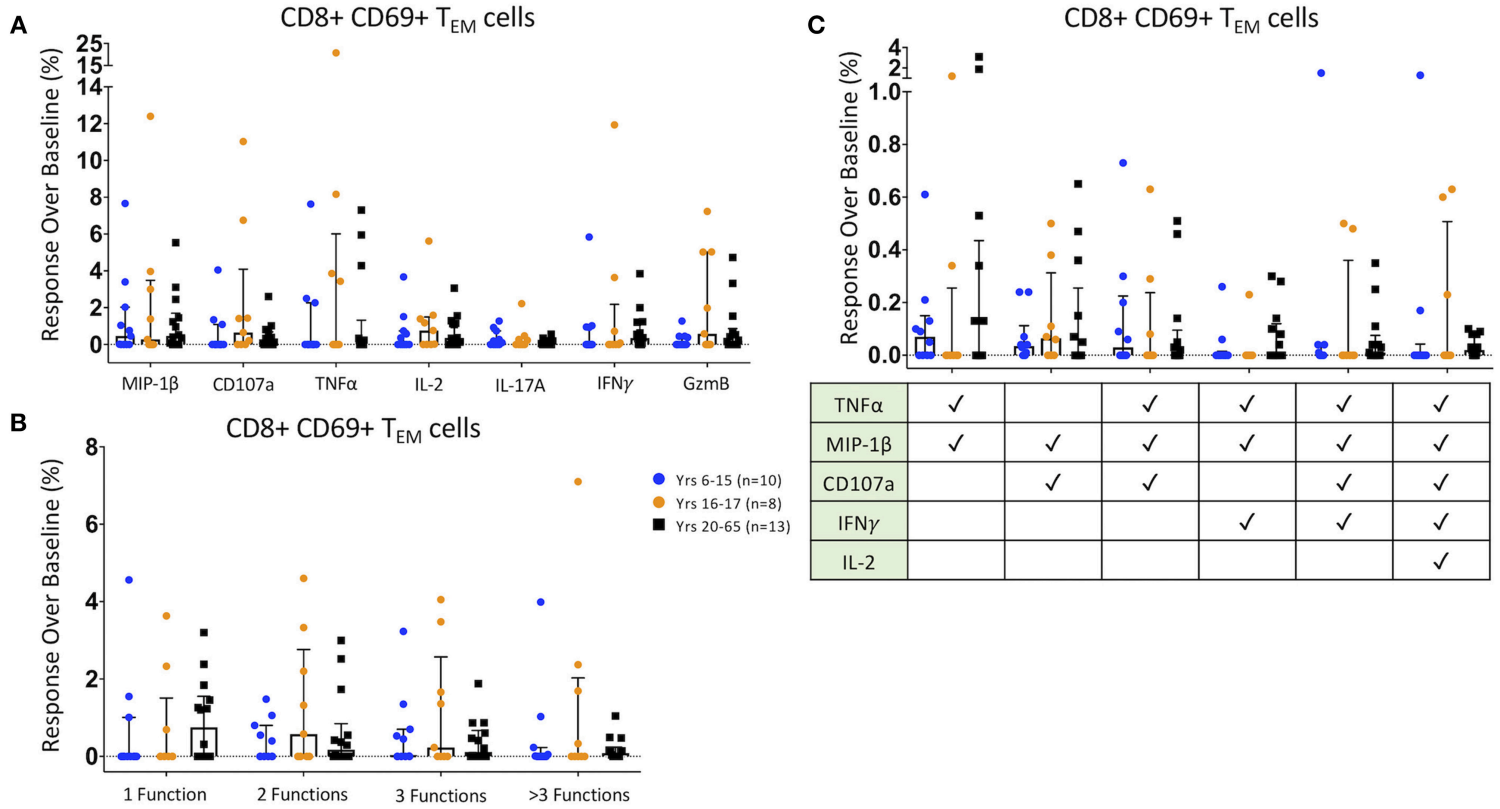
CD8+ T Effector Memory Responses. Scatter plots showing **(A)** the responses over baseline percentages of CD8+ CD69+ T_EM_ producing/expressing MIP1β, CD107a, TNFα, IL-2, IL-17A, IFNγ, and Granzyme B (GzmB); **(B)** CD8+ CD69+ T_EM_ observed mono-, bi-, tri-, and >3-functional populations; and **(C)**. specific CD8+ CD69+ T_EM_ selected multifunctional responses following co-culture with HLA-E restricted *S*. Typhi infected antigen presenting targets. These MF populations were selected based on those shown in a previous *S*. Typhi challenge study to be associated with protection from developing disease (10, 11). Bars represent medians with whiskers indicating interquartile ranges. Results are shown for the following populations: 6–15 year-old pediatric (*n* = 10), 16–17 year-old pediatric (*n* = 8), and adult (*n* = 13) participants. Scatter plot statistics were analyzed by unpaired *t*-test. (*p < 0.05).

We then analyzed the functionality of HLA-E-restricted antigen-responsive CD8^+^ T_EM_ cells. We identified increases in MIP-1β, CD107a, TNFα, IL-2, IL-17A, IFNγ, and Granzyme B post-Ty21a vaccination responses to HLA-E restricted *S*. Typhi antigen presentation over pre-vaccination responses (response over baseline) in many individual participants. There were no significant age-associated differences observed among individual effector functions (Figure 3A). Additionally, among observed mono- and multi-functional activation states -as defined by the increased expression of one or more of the aforementioned effector functions identified with the FCOM function in Winlist- the percentage of responses over baseline showed no significant overall age-associated differences (Figure 3B). Finally, we analyzed specific multifunctional responses over baseline in the HLA-E restricted co-culture model, which were shown in a previous *S*. Typhi challenge study to be associated with protection from developing, and/or delayed onset of, disease (10, 11). Among these six multifunctional populations, as identified with FCOM, there were no significant differences among age groups (Figure 3C).

Similarly, no significant differences among CD8+ T_EM_ effectors were observed when dividing the participants by sex or as age, although Granzyme B^+^ expression trended toward significance comparing pediatric and adult male participants to female pediatric participants (Supplemental Figure 3A). Interestingly, the observed monofunctional CD8^+^ T_EM_ population among adult males was significantly higher than either of the female age-groups (Supplemental Figure 3B), likely due to the observed higher Granzyme B expression in this group. Further, when comparing the specific, multifunctional populations associated with protection, we observed that males—especially pediatric males—were more likely to have higher percentages of HLA-E restricted *S*. Typhi-responsive CD107a/MIP-1β double-positive cells following vaccination than pediatric females (Supplemental Figure 3C). Among the HLA-E haplotype divided populations, no significant individual CD8^+^ T_EM_ effector responses-over-baseline were seen (Supplemental Figure 3D). HLA-E^*^01:03 exhibited significantly higher monofunctional responses than heterozygous participants (Supplemental Figure 3E), but there were no significant differences in Ty21a-induced multifunctional populations, including those which has been associated with protection in previous studies (10, 11) (Supplemental Figure 3F), among the HLA-E haplotypes.

Taken together, we observed few significant response-over-baseline trends associated with Ty21a vaccination among HLA-E restricted *S*. Typhi-responsive CD8^+^ T_EM_, based on traditional T_C_1 and T_C_17 effector molecules. The trends that were observed were likely due to the wide dispersion of the responses in individual volunteers seen within age groups, sex, and HLA-E haplotypes.

### CD8^+^ T_EMRA_ Responses Following Ty21a Vaccination

**Figure 4:**
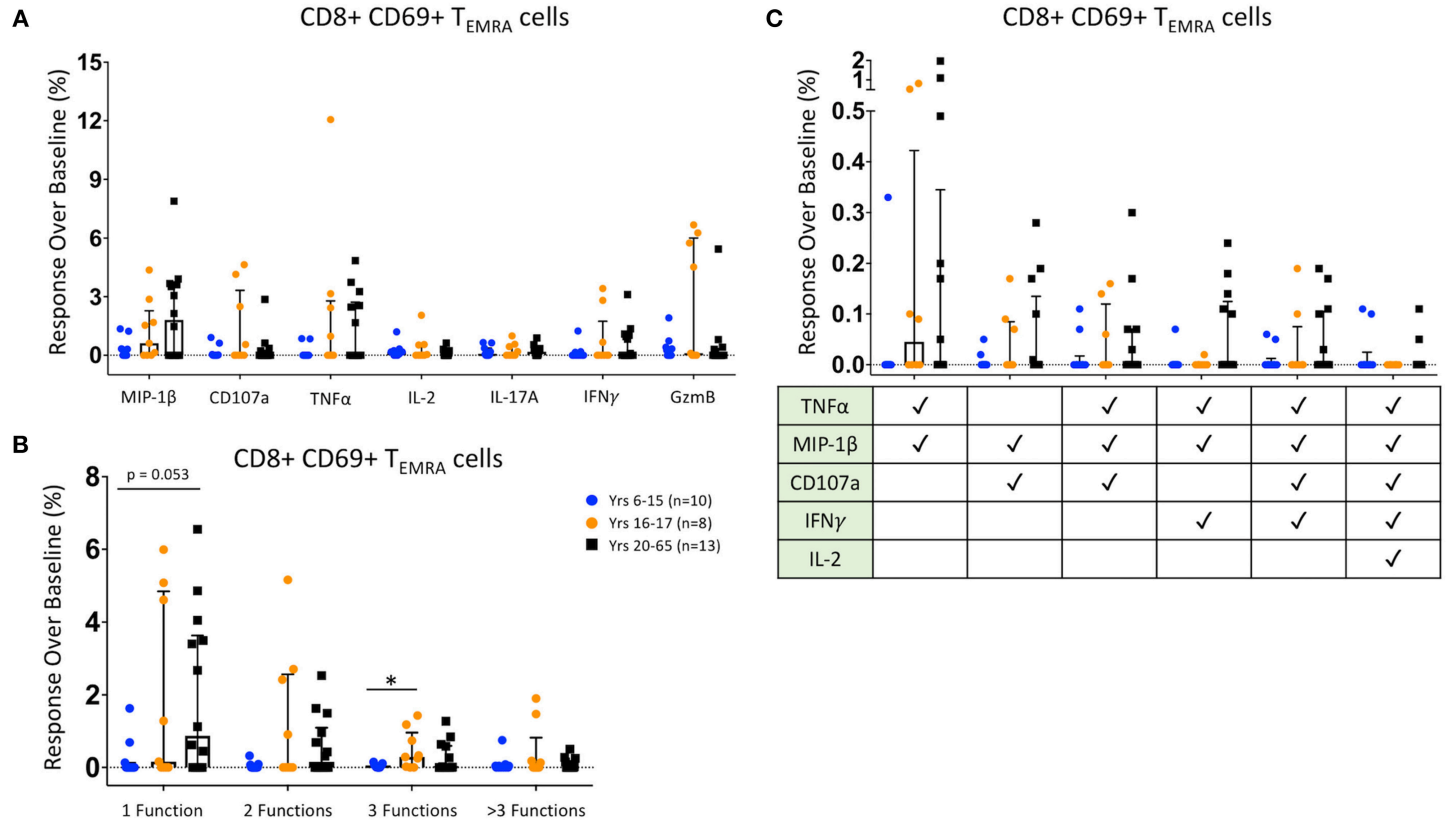
CD8+ T Effector Memory RA Responses. Scatter plots showing **(A)** the responses over baseline percentages of CD8+ CD69+ T_EMRA_ producing/expressing MIP1β, CD107a, TNFα, IL-2, IL-17A, IFNγ, and Granzyme B (GzmB); **(B)** CD8+ CD69+ T_EMRA_ observed mono-, bi-, tri-, and >3-functional populations; and **(C)**. specific CD8+ CD69+ T_EMRA_ selected multifunctional responses following co-culture with HLA-E restricted *S*. Typhi infected antigen presenting targets. These MF populations were selected based on those shown in a previous *S*. Typhi challenge study to be associated with protection from developing disease (10, 11). Bars represent medians with whiskers indicating interquartile ranges. Results are shown for the following populations: 6–15 year-old pediatric (*n* = 10), 16–17 year-old pediatric (*n* = 8), and adult (*n* = 13) participants. Scatter plot statistics were analyzed by unpaired *t*-test. (*p < 0.05).

We then analyzed the functionality of HLA-E-restricted antigen-responsive CD8^+^ T_EMRA_ cells. We identified increases over baseline of HLA-E-restricted CD8^+^ T_EMRA_ expression of MIP-1β, CD107a, TNFα, IL-2, IL-17A, IFNγ, and Granzyme B among many Ty21a-vaccinated participants. Interestingly, MIP-1β and TNFα responses were significantly higher among CD8^+^ T_EMRA_ from adults than in our younger pediatric participants, and Granzyme B expression was significantly higher in 16–17 year-old participants than among either adults or younger pediatric vaccines (Figure 4A). Further, when analyzing functional states, we found that younger pediatric vaccines show significantly fewer observed mono- and bi-functional CD8^+^ T_EMRA_ than both adult and older adolescent participants, while 16–17 year-old individuals show significantly greater tri-functional populations than the younger participants (Figure 4B). There were no significant differences across age groups in the induction of defined protection-associated multifunctional populations; however, adults trended toward a greater percentages of TNFα/MIP-1β/IFNγ triple-positive CD8^+^ T_EMRA_ than either pediatric group (Figure 4C).

As with the CD8^+^ effector memory cells, we divided our participant by sex and age. Adult males showed significantly greater percentages of MIP-1β and TNFα, and pediatric males showed significantly more Granzyme B expression, when compared to pediatric females (Supplemental Figure 4A). These low percentages of functional CD8^+^ T_EMRA_ in pediatric females was also seen in the analysis of observed mono- and multifunctional responses over baseline, where their monofunctional responses were significantly lower than adults of both genders, and their bifunctional responses were lower than adult males (Supplemental Figure 4B). Among defined protection-associated multifunctional populations, adult males have significantly higher percentages of TNFα/MIP-1β/IFNγ triple-positive CD8^+^ T_EMRA_ than both male and female pediatric participants (Supplemental Figure 4C). Further, the adult males also have significantly greater TNFα/MIP-1β/CD107a/IFNγ responsiveness than pediatric females (Supplemental Figure 4C). There were, however, no significantly distinct effector responses among the various HLA-E haplotypes (Supplemental Figure 4D). Both homozygous haplotypes showed greater monofunctional responses over baseline compared to the heterozygous participants (Supplemental Figure 4E). Among the specific multifunctional subsets, the HLA-E^*^01:03 quintuple-positive response was significantly higher than the heterozygous participants; however, the significance of these findings is unclear given the relatively small number of individuals in this cohort and the low magnitude of responses (Supplemental Figure 4F).

Taken together, the CD8^+^ T_EMRA_ response over baseline to HLA-E restricted *S*. Typhi-infected target cells are variable among individuals but in general show more significant age- and gender-associated differences than observed with T_EM_, particularly between adult males and pediatric females.

### Protection-Associated Multifunctional T Cells With Granzyme B Expression

The *S*. Typhi challenge study that found a correlation of certain multifunctional T cell responses and protection did not include Granzyme B expression (10, 11). However, because it is likely that the expression of cytotoxic Granzyme B would aid in defining the protective capabilities of multifunctional T cells, the current studies also included Granzyme B expression to complement the analyses of “protection-associated” multifunctional responses previously described for both CD69^+^CD8^+^ T_EM_ and T_EMRA_ (Supplemental Figure 5). Interestingly, the analyses of Granzyme B production identified significantly greater percentages of TNFα/MIP-1β/GzmB triple-positive by PBMC from adult participants, as well as TNFα/MIP-1β/CD107a/IFNγ/GzmB quintuple-positive T_EM_ cells in adult and 16–17 year-old pediatric participants than those observed in the 6–15 year-old participants (Supplemental Figure 5A). T_EMRA_ responsiveness shows fewer significant differences with the addition of Granzyme B than without (Supplemental Figure 5B). Similar to previous observations, the bulk of these differences appear to be between adult males and pediatric females, among T_EM_ (Supplemental Figure 5C) and T_EMRA_ (Supplemental Figure 5D). There were no significant differences between HLA-E haplotypes with Granzyme B-added to the study of T_EM_ multifunctional populations (Supplemental Figure 5E). However, HLA-E^*^01:01 participants showed a significantly higher percentage of TNFα/MIP-1β /IFNγ/GzmB quadruple-positive T_EMRA_ than heterozygous participants. Of note, no HLA-E^*^01:03 participants were considered vaccine responders (Supplemental Figure 5F).

### tSNE Analysis of Activated CD8^+^ T_EM_ Populations

**Figure 5:**
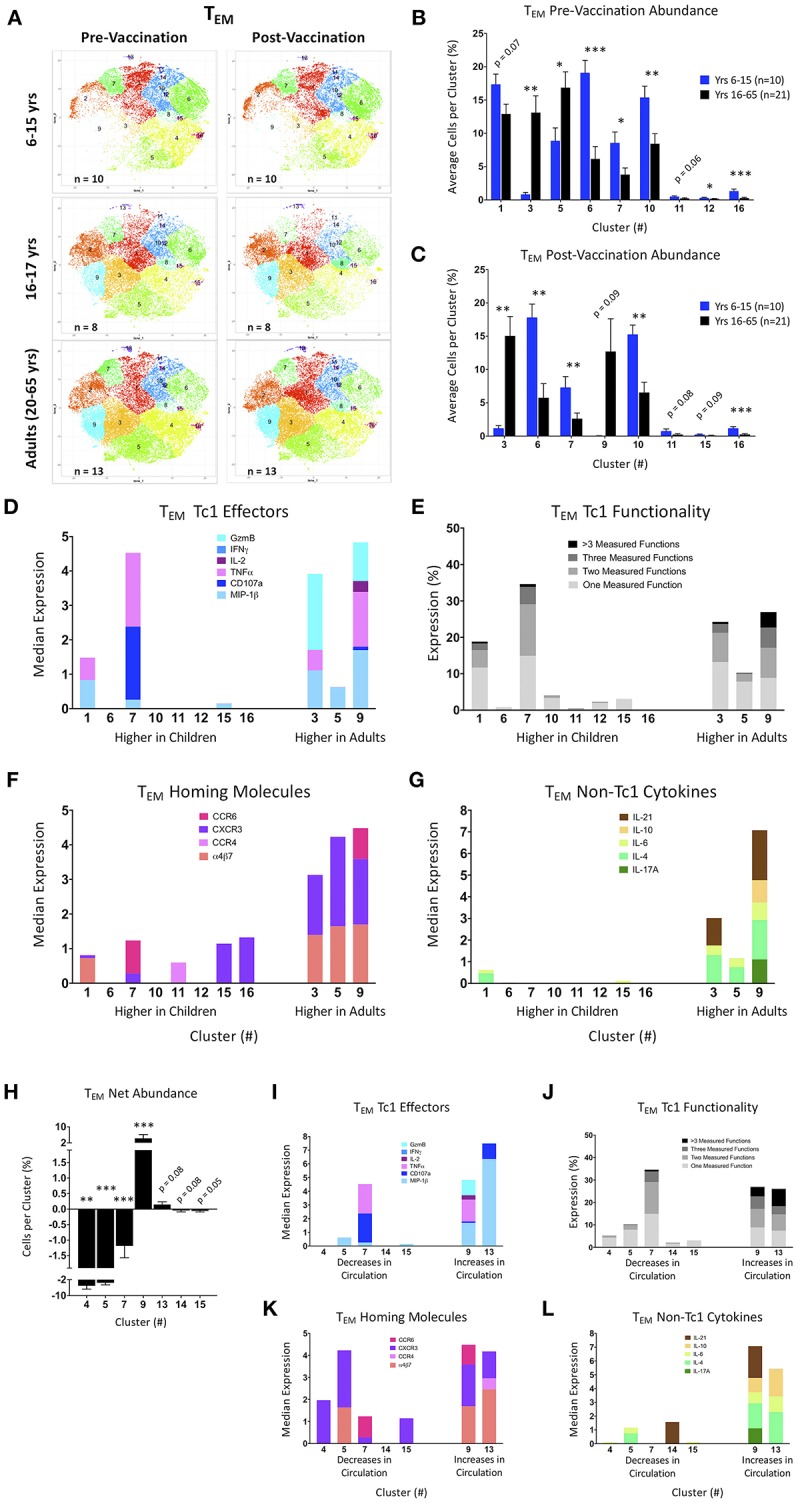
tSNE analysis of activated CD8+ T effector memory populations. **(A)** tSNE maps of pre- and post-Ty21a vaccinated 6–15 year old, 16–17 year old, and adult participants. **(B,C)** Significantly different clusters between age groups (6–15 year old and 16 years and over) represented by bar graphs identifying the average percent of cells per volunteer per cluster in pre-Ty21a **(B)** and postTy21a **(C)** immunization timepoints. **(D–G)** Functions of significantly different tSNE clusters from **(B,C)**, divided into: **(D)** median expression of Tc1 effector functions MIP1β, CD107a, TNFα, IL-2, IFNγ, and Granzyme B (GzmB). **(E)** Winlist FCOM-analysis determined Tc1 mono- and multifunctional cytokine expression. **(F)** median expression of homing markers, and **(G)** median expression of non-Tc1 associated cytokines. **(H)** Clusters showing significant net changes in the percent of cells following Ty21a vaccination. **(I–L)** Functions of significantly different tSNE clusters from **(H)**, divided into: **(I)**. median expression of Tc1 effector functions. MIP1β, CD107a, TNFα, IL-2, IFNγ, and Granzyme B (GzmB), **(J)** Winlist FCOM-analysis determined Tc1 mono- and multifunctional cytokine expression, **(K)** median expression of homing markers, and **(L)** median expression of non-Tc1 associated cytokines.

Activated (CD69^+^) CD8^+^ T_EM_ cell data were exported and processed on the Cytofkit package available in the R biocLite module. Sixteen clusters were identified following tSNE visualization using the ClusterX unbiased clustering method on the following markers: IL-4, CXCR5, α4β7, IL-6, CCR4, MIP-1β, CD107a, TNFα, IL-2, CXCR3, IL-17A, IFNγ, IL-10, CD154 (CD40L), Granzyme B, IL-21, ICOS, PD-1, and CCR6 (Supplemental Figure 6A). Individual participants were parsed into pre- and post-Ty21a vaccinated age groups (6–15, 16–17, and 20–65 years old) and cells were pooled into a single cluster analysis. To ensure that the proportions of the various clusters were representative of the various age and treatment groups, similar numbers of cells from each of the participants and time points were included in the tSNE analyses shown in Figure 5. Of note, the younger pediatric participants clearly showed much fewer cells in clusters 2, 3, 8, and 9 compared to the older pediatric and adults at both pre- and post-vaccine timepoints (Figure 5A). Importantly, older pediatric and adult tSNE populations are very similar. Thus, all downstream analysis was focused on comparing the responses of 6–15 year-old participants with those observed in 16–65 year-old participants.

Clusters were then analyzed based on the average percentage of cells per cluster, split by age group, and vaccination status (Figures 5B,C). Of note, clusters 11 through 16 have much fewer percentage of cells per cluster than clusters 1 through 10. Adults show significantly higher percentages of cells in clusters 3 and 5 pre-vaccination, whereas younger children have higher percentages of cells in clusters 6, 7, 10, 12, and 16 (Figure 5B). Following Ty21a vaccination, clusters 3 and 9 are significantly more abundant in adults, while clusters 6, 7, 10, and 16 remain significantly higher among younger children (Figure 5C). Interestingly, although the tSNE graphs show some clusters (e.g., 2 and 9) that appear different between age groups, they approached (*p* = 0.15), but did not reach statistically significant differences between groups, likely due to over-representation in these clusters of a few individuals. We then analyzed the median expression of conventional cytotoxic CD8^+^ T cell (T_C_1) effectors in each significant cluster, thus characterizing the phenotype of the cells within the clusters that are differentially represented among age groups. Through this analysis, it became clear that clusters 3, 7, and 9 are made up of cells capable of co-expressing several T_C_1 functions (Figure 5D). By gating and analyzing each cluster with FCOM in Winlist, we are able to identify the percentage of cells that express one or more of each of the six T_C_1 functions. This downstream supervised analysis identified clusters 3, 7, and 9, as those that contain the greatest percentage of multifunctional CD8^+^ CD69^+^ T_EM_ (Figure 5E). In addition to conventional T_C_1 effectors, we also studied the homing characteristics of the cells in each cluster. This analysis showed that clusters with significantly higher percentages of adult cells also contain cells with high median expression of the gut-homing molecule integrin α4β7, as well as inflammation-associated homing chemokine CXCR3 and, in the case of cluster 9, the mucosal-homing chemokine CCR6 (Figure 5F). We also explored the median expression of non-T_C_1 cytokines within our CD8^+^ CD69^+^ T_EM_ and found that multifunctional cluster 9 contained nearly all of the IL-17A producing cells, though cluster 3 was found, together with cluster 9, to produce the T_C_17-associated cytokine IL-21 (Figure 5G). Of note, both of these clusters are more represented by adult cells.

We next explored significant changes in cluster abundance following Ty21a vaccination by identifying the net change in the percentage of cells per cluster (Figure 5H). Of note, only 16–65 year old participants showed any significant differences in T_EM_ clusters following Ty21a vaccination. These significant clusters were then analyzed as in Figures 5D–G, here based on whether they increased or decreased from circulation following vaccination (Figures 5I–L). Cluster 9, which increases in adults following vaccination, is made up of multifunctional T_EM_, capable of expressing multiple Tc1 and Tc17 effectors, and a variety of homing molecules. With the exception of cluster 7, the clusters that decrease following Ty21a vaccination tend to contain T_EM_ that are less functional based on the effectors measured in these experiments.

We then performed supervised gating and FCOM analyses of the most multifunctional clusters to explore the percentages of the multifunctional populations associated with protection as described previously. Interestingly, cluster 2 seemed to be populated by many of the more protective phenotypes with or without Granzyme B included in the analyses (Supplemental Figures 7A,B), but cluster 9 is also representative of the Granzyme B-containing populations (Supplemental Figure 7B). In addition to T_C_1 effectors, non-T_C_1 effectors, and homing markers, we also explored expression of follicular-associated molecules such as CXCR5, activation molecules CD154 (CD40L) and ICOS, as well as the exhaustion/activation marker PD-1. Interestingly, ICOS [which has been shown to be important for maintenance of T_C_17 in mice (42)] was present in many clusters (Supplemental Figure 7C). Further, PD-1 was present in the highly multifunctional cluster 9, and CXCR5 is seen in the low-abundance multifunctional cluster 13. Finally, in addition to dividing by age, we also parsed activated CD8^+^ T_EM_ tSNE maps based on gender and HLA-E haplotype to determine the presence of Ty21a associated relationships. Marked differences were observed in the dominance of various clusters between males and females, whether pre- or post-vaccination (Supplemental Figure 7D). Of note, clusters 2, 3, 5, and 13 are more highly represented in males, whereas cluster 8 is overrepresented in females, many of those from the vaccinated pool (Supplemental Figures 7D,E). Marked differences were observed in the dominance of various clusters between unmatched HLA-E^*^01:03 and both HLA-E^*^01:01 and heterozygous, whether pre- or post-vaccination (Supplemental Figure 7F). Unmatched HLA-E^*^01:03 participants showed a low average number of cells in several clusters, particularly in the more multifunctional clusters (Supplemental Figures 7F,G).

### tSNE Analysis of Activated CD8^+^ T_EMRA_ Populations

**Figure 6:**
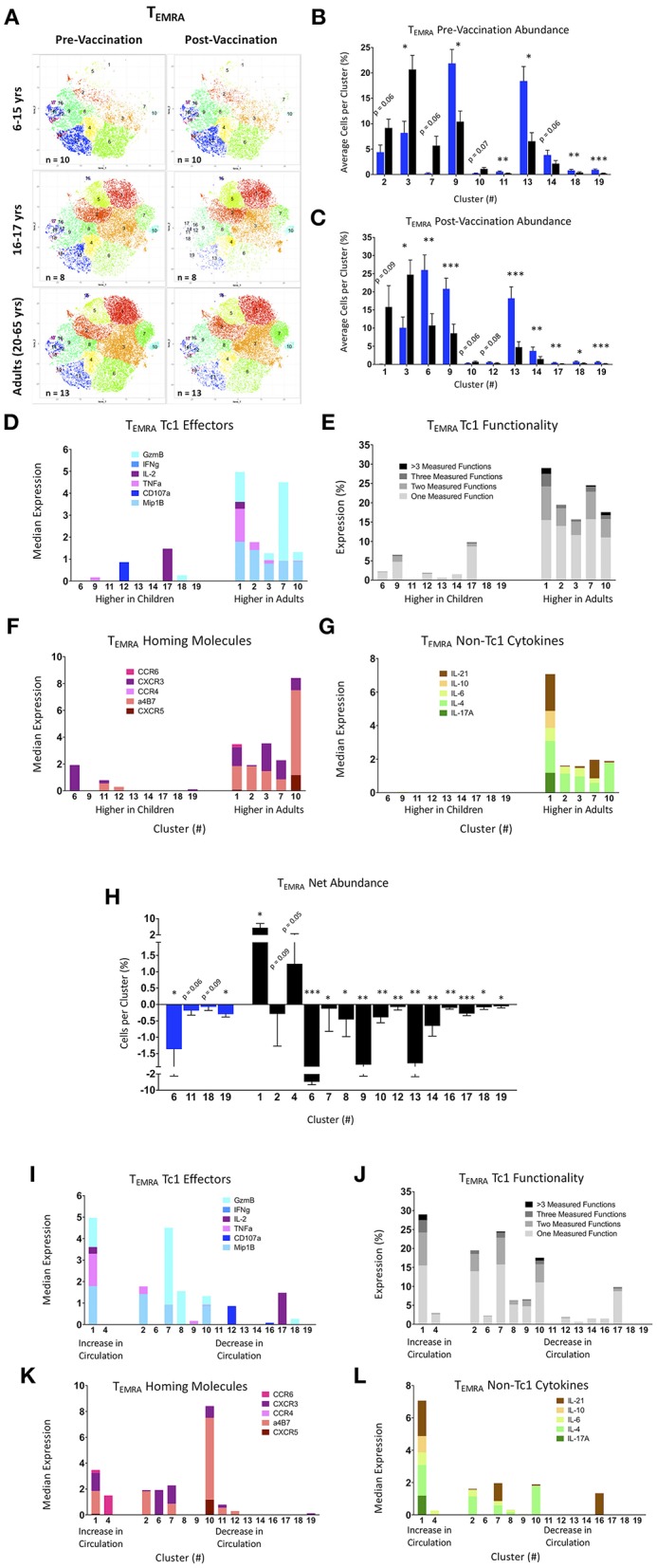
tSNE analysis of activated CD8+ T effector memory RA (T_EMRA_) populations. **(A)** tSNE maps of pre- and post-Ty21a vaccinated 6–15 year old, 16–17 year old, and adult participants. **(B,C)** Significantly different clusters between age groups (6–15 year old and 16 years and over) represented by bar graphs identifying the average percent of cells per volunteer per cluster in pre-Ty21a **(B)** and post-Ty21a **(C)** immunization timepoints. **(D–G)** Functions of significantly different tSNE clusters from **(B,C)**, divided into: **(D)** median expression of Tel effector functions MIP1β, CD107a, TNFα, IL-2, IFNγ, and Granzyme B (GzmB), **(E)** Winlist FCOM-analysis determined Tc1 mono- and multifunctional cytokine expression, **(F)** median expression of homing markers, and **(G)** median expression of non-Tc1 associated cytokines. **(H)** Clusters showing significant net changes in the percent of cells following Ty21a vaccination. **(I–L)** Functions of significantly different tSNE clusters from **(H)**, divided into: **(I)** median expression of Tc1 effector functions MIP1β, CD107a, TNFα, IL-2, IFNγ, and Granzyme B (GzmB), **(J)** Winlist FCOM-analysis determined Tc1 mono- and multifunctional cytokine expression, **(K)** median expression of homing markers, and **(L)** median expression of non-Tc1 associated cytokines.

Activated (CD69^+^) CD8^+^ T_EMRA_ were analyzed using the same methods and clustering markers as utilized for the T_EM_ data above (Supplemental Figure 6B) and generated 19 distinct clusters. To ensure that the proportions of the various clusters were representative of the various age and treatment groups, similar numbers of cells from each of the participants and time points were included in the tSNE analyses shown in Figure 6. The pre- and post-vaccine tSNE maps of younger pediatric participants are rather sparse compared with the older pediatric and adult maps, particularly within clusters 1, 2, 3, 5, and 7 (Figure 6A). Again, due to their similarity, 16–17 year-olds were combined with adults for downstream comparisons to the 6–15 year-old participants.

As with the T_EM_, clusters were analyzed based on average percentage of cells per cluster, split between age group, and vaccination status (Figures 6B,C). Here, clusters 10 through 12, and 15 through 19 have much fewer percentage of cells per cluster than clusters 1 through 9, 13, and 14. Among the pre-Ty21a time-point, clusters 9, 11, 13, 18, and 19 are significantly more abundant in children, while only cluster 3 is significantly higher in adults (with clusters 7 and 10 trending toward significance; Figure 6B). Following Ty21a vaccination, clusters 6, 9, 13, 14, 17, 18, and 19 are all made up of a significantly higher percentage of pediatric T_EMRA_, while cluster 3 again is the only population significantly higher in adults (with clusters 1 and 10 trending toward significance; Figure 6C). As discussed above regarding the T_EM_ data, although the tSNE graphs show striking differences in certain clusters (e.g., 1 and 5), only trends which did reach statistical significance (*p* < 0.05), or trend toward significance (*p* < 0.1), were observed for differences in these clusters between age groups. Only post-Ty21a cluster 1 exhibited a close trend toward a significant difference (*p* = 0.09). We then analyzed the median expression of conventional T_C_1 effectors in each cluster. We observed that clusters made up mostly of adult T_EMRA_ are more diverse (Figure 6D), and more multifunctional, as determined by FCOM analyses (Figure 6E). Exploration of the median expression of homing molecules show that the more multifunctional clusters more abundant in adults contain cells capable of homing to the gut (integrin α4β7 expression) and sites of inflammation (CXCR3 expression; Figure 6F). Further, T_EMRA_ capable of producing non-Tc1 cytokines, including cluster 1 which contains cells capable of producing IL-17A, previously reported to play an important role in protection against *S*. Typhi, are only seen in clusters higher in adults (Figure 6G) (10, 11, 32). Interestingly, clusters that are more frequently observed in younger participants show little-to-no median expression of T_C_1 effectors, gut-homing molecules, or non-T_C_1 cytokines.

We also explored significant changes in cluster abundance following Ty21a vaccination by identifying net changes in the percentage of cells per cluster (Figure 6H). Interestingly, only two clusters significantly increased in circulation following vaccination, and those were only found 16–65 year old participants. The significant clusters were analyzed based on Tc1 effector, Tc1 multifunctionality, homing, and non-Tc1 cytokines, here based on whether they increased or decreased from circulation following vaccination (Figures 6I–L). Cluster 1, which increases in adults following vaccination, is capable of expressing multiple Tc1 and Tc17 effectors, integrin α4β7, and CXCR3, and shows high multifunctional potential. The T_EMRA_ clusters that decrease following Ty21a vaccination are more variable in their functional and homing characteristics, but are not of the same magnitude as seen in cluster 1.

Similar to the T_EM_ analysis, we explored multifunctional populations which have been associated with protection, and identified cluster 5 as containing the greatest percentage of “protective phenotypes” with and without the addition of Granzyme B as one of the parameters (Supplemental Figures 8A,B). Additionally, we explored non-T_C_1 effectors expression of CXCR5, CD154 (CD40L), ICOS, and PD-1 among our CD8^+^ CD69^+^ T_EMRA_. ICOS was less frequently observed among T_EMRA_ than among T_EM_, but it was present in multifunctional clusters 1 and 15 (Supplemental Figure 8C). Exhaustion and activation marker PD-1 is also abundant within cluster 1. Interestingly, cluster 10 contains nearly all of the CXCR5 observed among the T_EMRA_, a very small cluster by number of cells, present mostly in older adolescents and adults, that also contain cells expressing MIP1-β, IL-4, and abundant integrin α4β7 (Figures 6A–F and Supplemental Figure 8A). In the analyses of the T_EMRA_ tSNE maps divided by gender, we observed that females have fewer numbers of cells than males in multifunctional clusters 2, 3, and 5 (Supplemental Figures 8D,E). Finally, we observed that mismatched HLA-E^*^01:03 participants had a very low average number of cells for many clusters, especially multifunctional clusters 1, 2, 3, and 5 (Supplemental Figures 8F,G).

## Discussion

The oral live-attenuated Ty21a *S*. Typhi vaccine protects against the development of typhoid disease, at least in part, by inducing robust T cell responses. Challenge studies with wild-type *S*. Typhi have shown that *S*. Typhi-antigen-responsive HLA-E-restricted CD8^+^ T cells are associated with protection from, and/or delayed onset of, typhoid disease (10, 11), and thus are likely important for effective vaccination. However, there is no information on the function and magnitude of these restricted CD8^+^ T cells within pediatric Ty21a recipients. In this study we compared pediatric and adult *S*. Typhi-antigen-responsive HLA-E-restricted CD8+ T cells, pre- and post- Ty21a vaccination, to better understand the impact of age and gender on the immunity elicited by Ty21a vaccination.

This study confirms and extends previous observation by us and others that children have a lower proportion of CD8^+^ T_EM_ cells than adults, while the proportions of CD8^+^ T_EMRA_ show fewer age dependent traits (15–17, 19, 20). Interestingly, by dividing our participants by sex as well as age, we show that adult males have the greatest proportion of CD8^+^ T_EM_, particularly compared to pediatric males and females. Further, we show that Ty21a vaccination does not significantly impact the overall proportions of total T cells, CD8^+^ T cells, CD8^+^ T_EM_, or CD8^+^ T_EMRA_, regardless of age, sex, or HLA-E haplotype.

CD69 expression increases following T cell receptor (TCR) and costimulatory molecule engagement, and it is thereby a useful marker to explore T cell activation. In this study, we show that unstimulated cells from adults, particularly adult males, have higher baseline percentages of CD69^+^ CD8^+^ T cells than children in the absence of *in vitro* stimulation. These percentages do not change in Ty21a vaccinated individuals. Further, when looking at CD8^+^ T cell activation over baseline following *in vitro* stimulation with a *S*. Typhi-infected cell line expressing HLA-E in the absence of classical class Ia HLA molecules, the global response to vaccination is observed in variable numbers of individuals, and the responses are of varying magnitude, independently of age or sex. Both HLA-E^*^01:01 and heterozygous participants showed baseline activation following *S*. Typhi antigen presentation, possibly the result of previous exposure to HLA-E-restricted activation of cross-reactive antigens. In contrast, HLA-E^*^01:03 participants had no positive CD69^+^ CD8^+^ T cell responses at baseline, likely due to their mismatch with the HLA-E^*^01:01-homozygous B-LCL cell line used. However, nearly all Ty21a-vaccinated HLA-E^*^01:03 participants showed positive activated CD8^+^ T cells after vaccination, suggesting the *S*. Typhi-infected targets express antigens/peptides which can be presented by both HLA-E^*^01:01 and HLA-E^*^01:03 molecules. Interestingly, a recent publication showed distinct peptide repertoires between the HLA-E allelic variants (43), although these studies did not explore the *S*. Typhi peptides shown in our lab to mediate at least some of the *S*. Typhi-HLA-E-restricted responses (30). Further studies will be needed to fully understand these observations.

Previous work from our lab has shown that *S*. Typhi-responsive CMI, including HLA-E-restricted T CMI, might play an important role in protection from typhoid disease (10, 11, 30–32). Further, multiple studies have shown that multifunctional T cell responses are critical for protection against pathogens, including S. Typhi (10, 11, 44–46). Thus, we investigated how Ty21a vaccination influences CD8^+^ T_EM_ and T_EMRA_ responses to *S*. Typhi-infected HLA-E restricted targets across age-groups, sex, and among HLA-E mismatched recipients. Our data show that CD8^+^ T_EM_ responses over baseline are variable among all groups, with respect to individual effector molecules (e.g., IFN-γ, TNF-α) and multifunctionality. However, in general, CD8^+^ T_EMRA_ responses over baseline tend to be stronger in adults than children, particularly in adult males. These data broaden our understanding of how Ty21a vaccination influences T cell responses depending on the age and sex of the individuals through a conserved means of antigen presentation. However, the variability among individuals suggested that conventional “supervised” analyses of only a single or a handful of activation molecules at a time might not be sufficient to uncover the complex immune responses at play. Thus, we developed a 35+ parameter mass cytometry panel to explore in depth the fine granularity of the HLA-E-restricted immune responses elicited by Ty21a immunization. However, it was not possible to evaluate such a complex set of key phenotypic, activation, and homing molecular markers with conventional flow cytometry analytical tools. This prompted us to use dimensionality reduction tools in an “unsupervised” fashion to further dissect these responses. Specifically, here we utilized tSNE to identify multifunctional populations within activated (CD69^+^) CD8^+^ T_EM_ and T_EMRA_ subsets and to extrapolate their pre- and post-Ty21a immunization relative abundance across age, sex, and HLA-E haplotype.

These results showed that highly multifunctional, gut-homing clusters are more frequently populated by adults and older pediatric participants, regardless of vaccination state. Of note, the follicular homing molecule CXCR5, a phenotypic marker used to identify circulating T follicular/helper cells (cT_FH_), was present in very few clusters despite recent studies identifying CXCR5+ CD8+ T cells to be of interest in other intracellular infections (47). This may be due to the previously observed early 3–7 day peak seen in the appearance of cT_FH_ in circulation following exposure to infectious organisms or vaccination (36), whilst in our studies PBMC were collected 14–42 days after Ty21a immunization. Alternatively, or in addition, CD8+ T_FH_ might be less responsive to bacterial pathogens -even intracellular pathogens such as *S*. Typhi. Future investigations will be required to address these possibilities. Further, we confirmed the observations utilizing conventional gating of T_EMRA_ responses; namely that these cells are less functional in circulation in younger children. Finally, using downstream gating of both T memory subsets, we observed that phenotypes previously described to be associated with protection from the development of typhoid disease are present in the most multifunctional clusters, particularly among those most abundant in adults and older pediatric participants. Interestingly, many of the clusters identified by tSNE seem to decrease in abundance following Ty21a vaccination. Previous work from our laboratory has shown fluctuations in circulating effector T cells following *S*. Typhi challenge or Ty21a immunization, likely due to homing and accumulation in the gut followed by a rebound of *S*. Typhi-specific cells in circulation (10, 11, 32), but the kinetics and magnitude of a potential homing to the gut in children remains unclear. This is largely due to the cell number limitations related to the difficulty in obtaining serial blood draws in this population. tSNE analysis allowed us to identify differences among groups that would otherwise have been very difficult, if not impossible to uncover. In fact, our results provide additional data in support of the contention that dimensionality reduction tools will be ever more critical in future studies as high multiparametric data becomes more extensive and commonplace.

Taken together, these data show that post-Ty21a *S*. Typhi-antigen-responsive HLA-E-restricted CD8^+^ T_EMRA_ show the greatest differences between age groups, with adults and older pediatric recipients exhibiting higher percentages of multifunctional cells as observed by both conventional gating and tSNE analysis. These results suggest the possibility of a decrease in efficacy among children vaccinated with Ty21a; however, the limited scope of this antigen presentation system merits further investigation into canonical classical class Ia MHC-restricted *S*. Typhi antigen presentation models among pediatric vaccinees. The present observations of decreased multifunctional T-cell potential in children, present until late teenage years, could prove of great importance in the development of new, effective oral live attenuated vaccines for use in pediatric populations.

## Author Contributions

MR, MM, and MS designed the study, analyzed the data, and wrote the manuscript. MR performed the experiments. WC and RB contributed to the design, collected and processed the clinical samples, and helped draft the manuscript. LM provided statistical expertise.

### Conflict of Interest Statement

The authors declare that the research was conducted in the absence of any commercial or financial relationships that could be construed as a potential conflict of interest.
