# Supplementary material for: Age-Associated Heterogeneity of Ty21a-Induced T Cell Responses to HLA-E Restricted Salmonella Typhi Antigen Presentation
Source: Front Immunol. 2019 Mar 4;10:257. doi: 10.3389/fimmu.2019.00257 (PMC6409365; doi:10.3389/fimmu.2019.00257)
Supplement: Supplementary file 1 [file Presentation_1.pptx]

## Slide 1
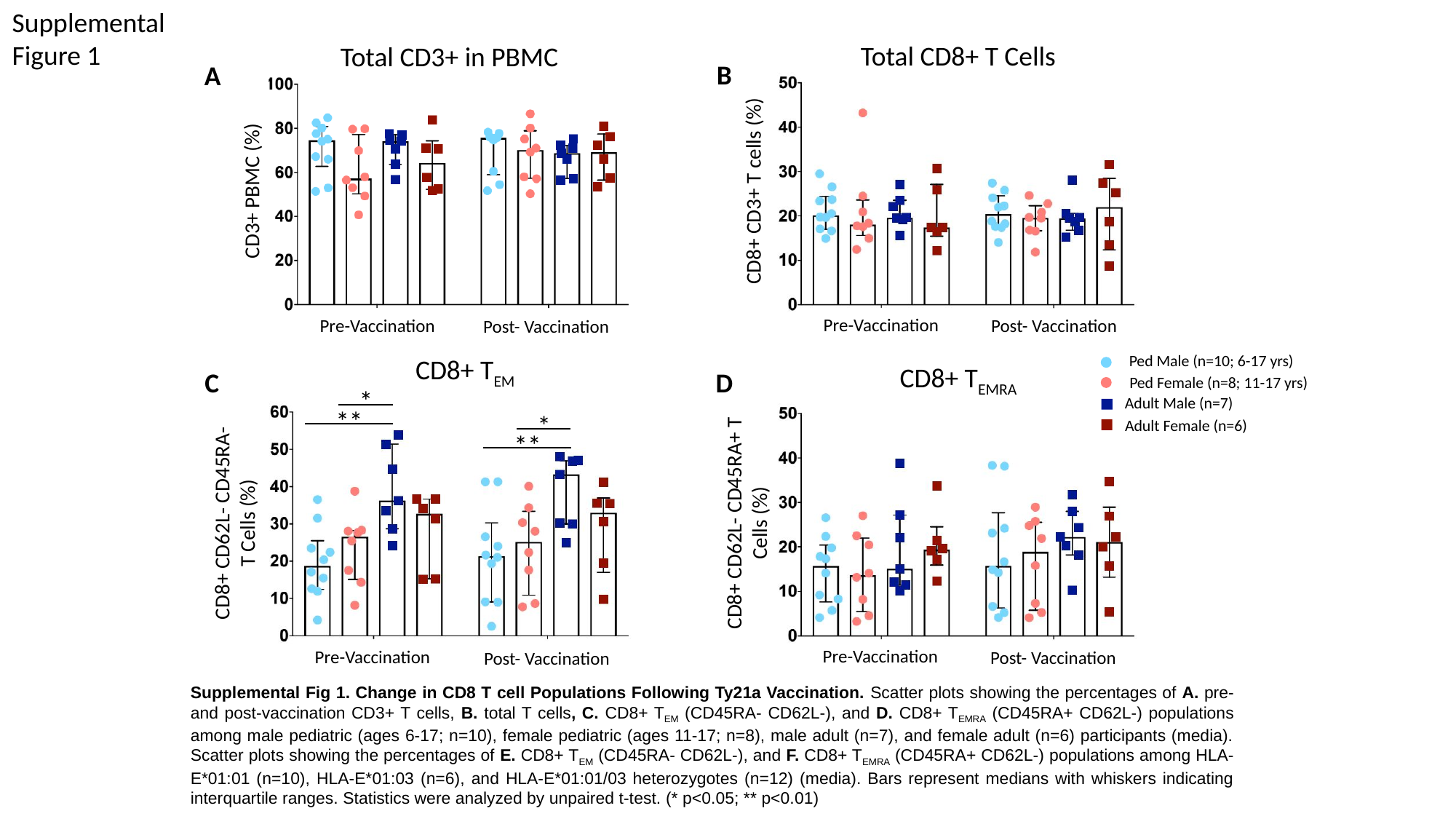

Supplemental Figure 1
Total CD8+ T Cells
Total CD3+ in PBMC
B
A
CD3+ PBMC (%)
CD8+ CD3+ T cells (%)
Pre-Vaccination
Pre-Vaccination
Post- Vaccination
Post- Vaccination
Ped Male (n=10; 6-17 yrs)
Ped Female (n=8; 11-17 yrs)
Adult Male (n=7)
Adult Female (n=6)
CD8+ TEM
CD8+ TEMRA
C
D
*
**
*
**
CD8+ CD62L- CD45RA- T Cells (%)
CD8+ CD62L- CD45RA+ T Cells (%)
Pre-Vaccination
Pre-Vaccination
Post- Vaccination
Post- Vaccination
Supplemental Fig 1. Change in CD8 T cell Populations Following Ty21a Vaccination. Scatter plots showing the percentages of A. pre- and post-vaccination CD3+ T cells, B. total T cells, C. CD8+ TEM (CD45RA- CD62L-), and D. CD8+ TEMRA (CD45RA+ CD62L-) populations among male pediatric (ages 6-17; n=10), female pediatric (ages 11-17; n=8), male adult (n=7), and female adult (n=6) participants (media). Scatter plots showing the percentages of E. CD8+ TEM (CD45RA- CD62L-), and F. CD8+ TEMRA (CD45RA+ CD62L-) populations among HLA-E*01:01 (n=10), HLA-E*01:03 (n=6), and HLA-E*01:01/03 heterozygotes (n=12) (media). Bars represent medians with whiskers indicating interquartile ranges. Statistics were analyzed by unpaired t-test. (* p<0.05; ** p<0.01)

## Slide 2
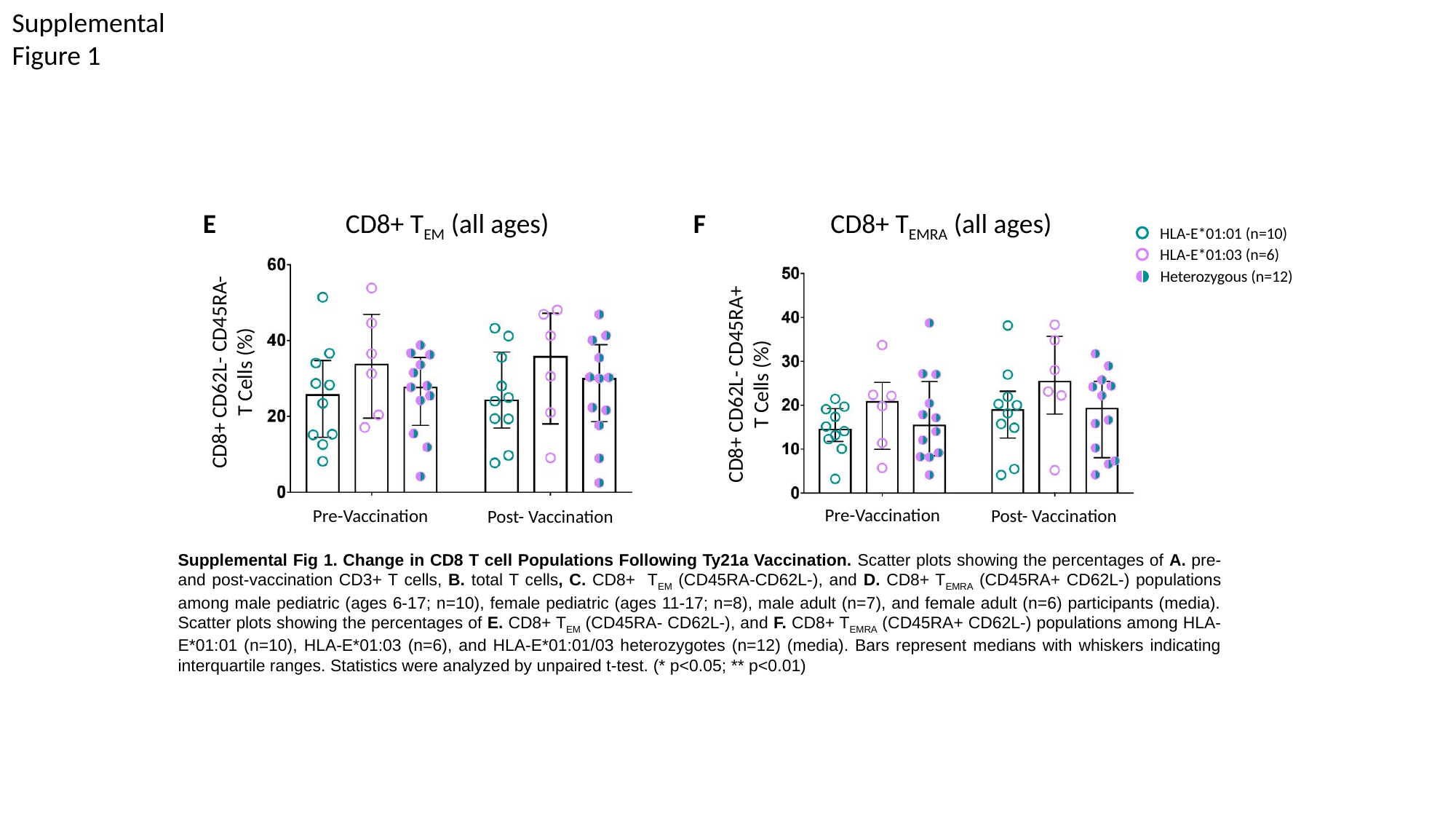

Supplemental Figure 1
E
CD8+ TEM (all ages)
CD8+ TEMRA (all ages)
F
HLA-E*01:01 (n=10)
HLA-E*01:03 (n=6)
Heterozygous (n=12)
CD8+ CD62L- CD45RA- T Cells (%)
CD8+ CD62L- CD45RA+ T Cells (%)
Pre-Vaccination
Pre-Vaccination
Post- Vaccination
Post- Vaccination
Supplemental Fig 1. Change in CD8 T cell Populations Following Ty21a Vaccination. Scatter plots showing the percentages of A. pre- and post-vaccination CD3+ T cells, B. total T cells, C. CD8+ TEM (CD45RA-CD62L-), and D. CD8+ TEMRA (CD45RA+ CD62L-) populations among male pediatric (ages 6-17; n=10), female pediatric (ages 11-17; n=8), male adult (n=7), and female adult (n=6) participants (media). Scatter plots showing the percentages of E. CD8+ TEM (CD45RA- CD62L-), and F. CD8+ TEMRA (CD45RA+ CD62L-) populations among HLA-E*01:01 (n=10), HLA-E*01:03 (n=6), and HLA-E*01:01/03 heterozygotes (n=12) (media). Bars represent medians with whiskers indicating interquartile ranges. Statistics were analyzed by unpaired t-test. (* p<0.05; ** p<0.01)

## Slide 3
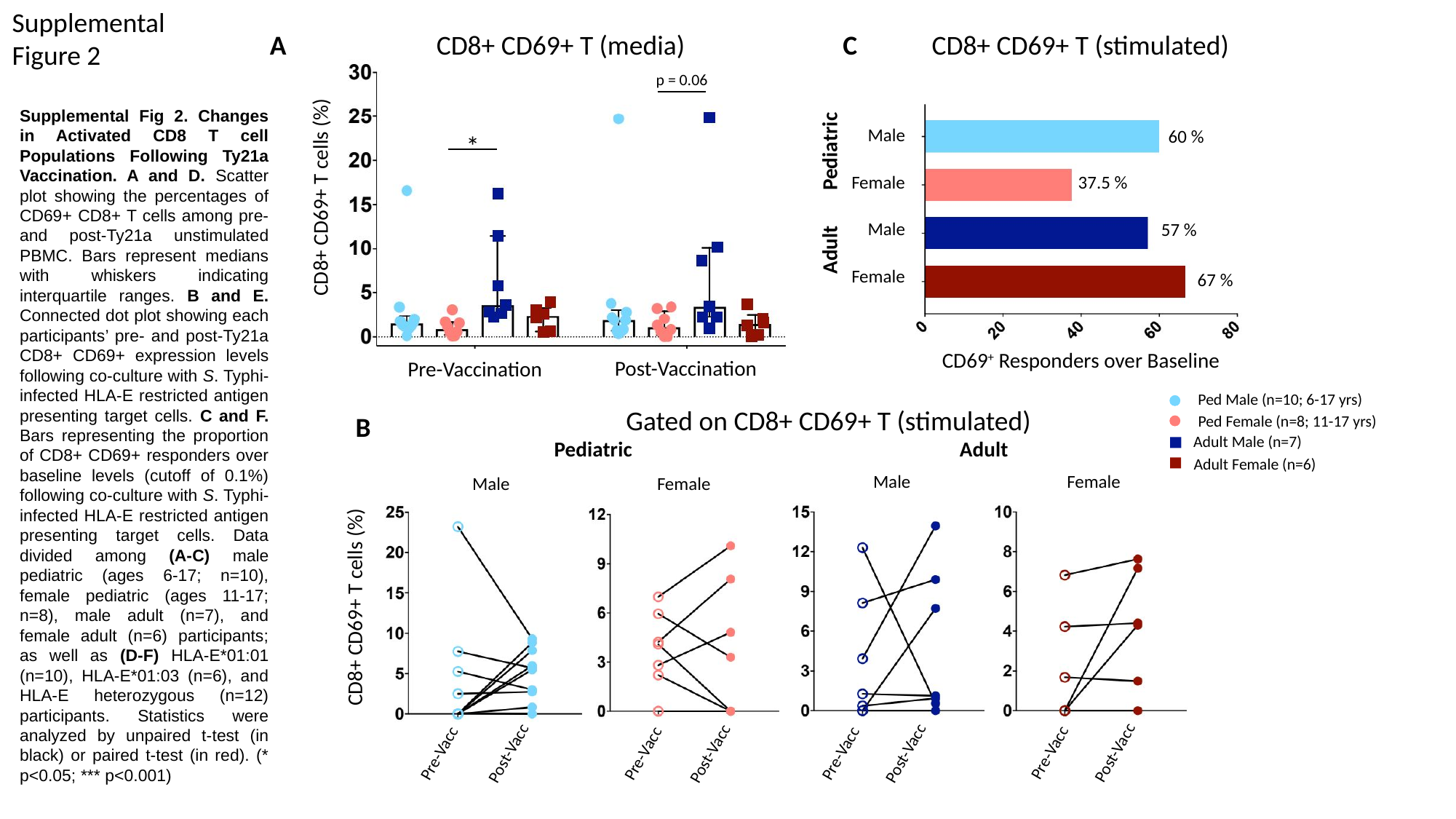

Supplemental Figure 2
C
A
CD8+ CD69+ T (stimulated)
CD8+ CD69+ T (media)
p = 0.06
Supplemental Fig 2. Changes in Activated CD8 T cell Populations Following Ty21a Vaccination. A and D. Scatter plot showing the percentages of CD69+ CD8+ T cells among pre- and post-Ty21a unstimulated PBMC. Bars represent medians with whiskers indicating interquartile ranges. B and E. Connected dot plot showing each participants’ pre- and post-Ty21a CD8+ CD69+ expression levels following co-culture with S. Typhi-infected HLA-E restricted antigen presenting target cells. C and F. Bars representing the proportion of CD8+ CD69+ responders over baseline levels (cutoff of 0.1%) following co-culture with S. Typhi- infected HLA-E restricted antigen presenting target cells. Data divided among (A-C) male pediatric (ages 6-17; n=10), female pediatric (ages 11-17; n=8), male adult (n=7), and female adult (n=6) participants; as well as (D-F) HLA-E*01:01 (n=10), HLA-E*01:03 (n=6), and HLA-E heterozygous (n=12) participants. Statistics were analyzed by unpaired t-test (in black) or paired t-test (in red). (* p<0.05; *** p<0.001)
Male
60 %
*
Pediatric
Female
37.5 %
CD8+ CD69+ T cells (%)
Male
57 %
Adult
Female
67 %
CD69+ Responders over Baseline
Post-Vaccination
Pre-Vaccination
Ped Male (n=10; 6-17 yrs)
Ped Female (n=8; 11-17 yrs)
Adult Male (n=7)
Adult Female (n=6)
Gated on CD8+ CD69+ T (stimulated)
B
Adult
Pediatric
Female
Male
Female
Male
CD8+ CD69+ T cells (%)
Pre-Vacc
Post-Vacc
Post-Vacc
Pre-Vacc
Pre-Vacc
Post-Vacc
Pre-Vacc
Post-Vacc

## Slide 4
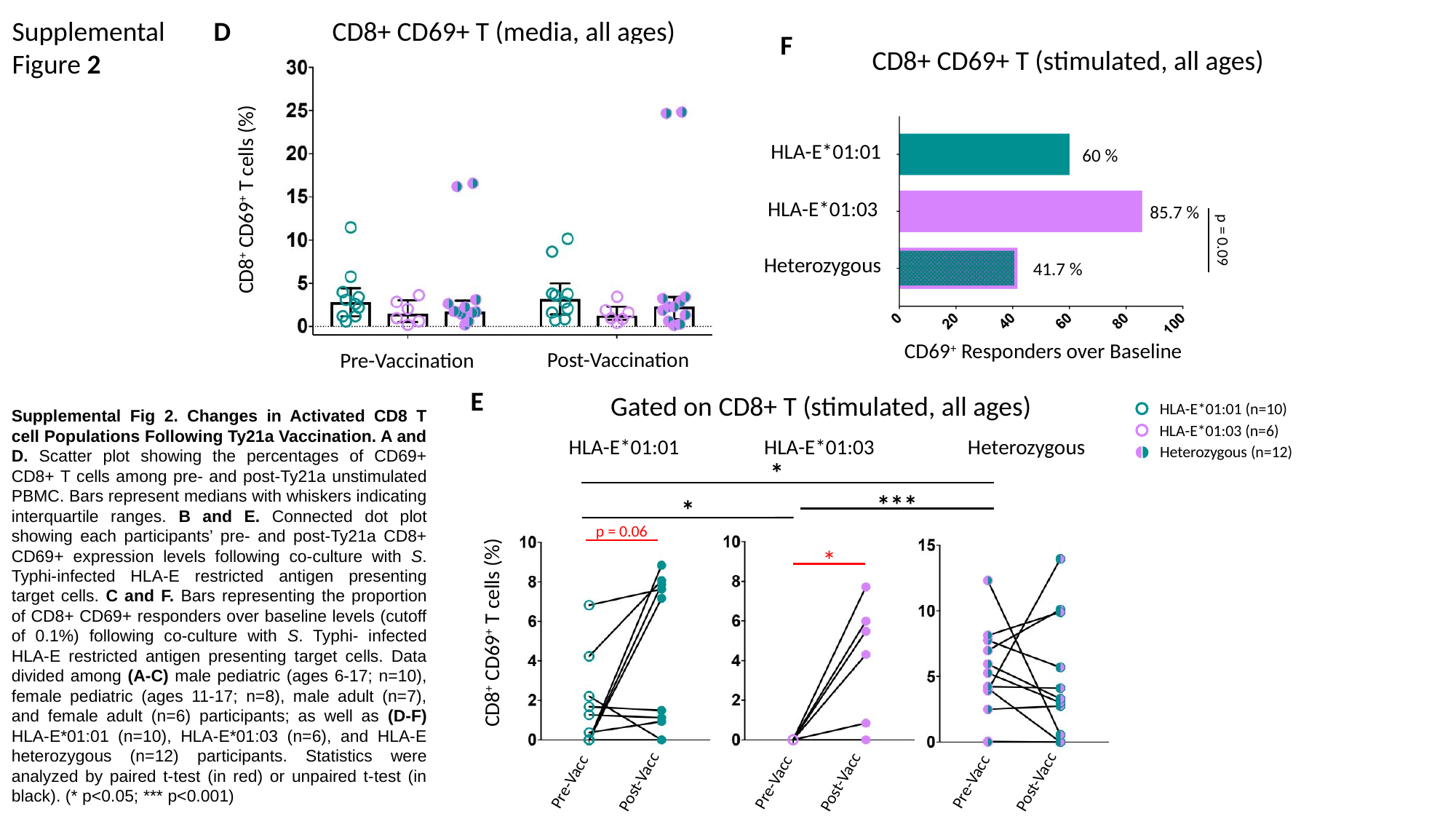

Supplemental Figure 2
D
CD8+ CD69+ T (media, all ages)
F
CD8+ CD69+ T (stimulated, all ages)
HLA-E*01:01
60 %
CD8+ CD69+ T cells (%)
HLA-E*01:03
85.7 %
p = 0.09
Heterozygous
41.7 %
CD69+ Responders over Baseline
Post-Vaccination
Pre-Vaccination
E
Gated on CD8+ T (stimulated, all ages)
HLA-E*01:01 (n=10)
HLA-E*01:03 (n=6)
Heterozygous (n=12)
Supplemental Fig 2. Changes in Activated CD8 T cell Populations Following Ty21a Vaccination. A and D. Scatter plot showing the percentages of CD69+ CD8+ T cells among pre- and post-Ty21a unstimulated PBMC. Bars represent medians with whiskers indicating interquartile ranges. B and E. Connected dot plot showing each participants’ pre- and post-Ty21a CD8+ CD69+ expression levels following co-culture with S. Typhi-infected HLA-E restricted antigen presenting target cells. C and F. Bars representing the proportion of CD8+ CD69+ responders over baseline levels (cutoff of 0.1%) following co-culture with S. Typhi- infected HLA-E restricted antigen presenting target cells. Data divided among (A-C) male pediatric (ages 6-17; n=10), female pediatric (ages 11-17; n=8), male adult (n=7), and female adult (n=6) participants; as well as (D-F) HLA-E*01:01 (n=10), HLA-E*01:03 (n=6), and HLA-E heterozygous (n=12) participants. Statistics were analyzed by paired t-test (in red) or unpaired t-test (in black). (* p<0.05; *** p<0.001)
HLA-E*01:03
HLA-E*01:01
Heterozygous
*
***
*
p = 0.06
*
CD8+ CD69+ T cells (%)
Post-Vacc
Pre-Vacc
Pre-Vacc
Post-Vacc
Pre-Vacc
Post-Vacc

## Slide 5
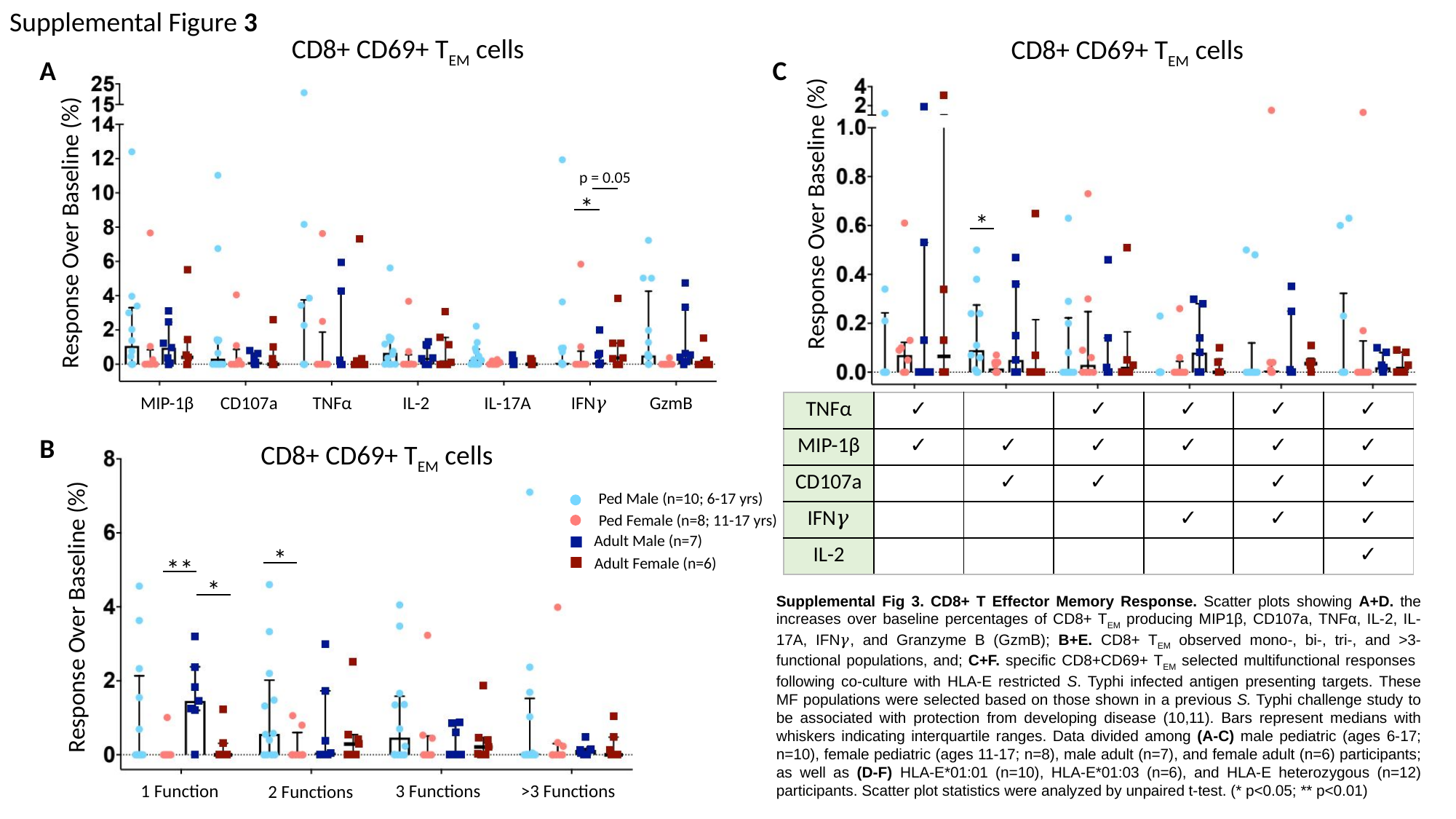

Supplemental Figure 3
CD8+ CD69+ TEM cells
CD8+ CD69+ TEM cells
A
C
p = 0.05
*
Response Over Baseline (%)
*
Response Over Baseline (%)
GzmB
IL-17A
IFN𝛾
TNFα
MIP-1β
IL-2
CD107a
| TNFα | ✓ | | ✓ | ✓ | ✓ | ✓ |
| --- | --- | --- | --- | --- | --- | --- |
| MIP-1β | ✓ | ✓ | ✓ | ✓ | ✓ | ✓ |
| CD107a | | ✓ | ✓ | | ✓ | ✓ |
| IFN𝛾 | | | | ✓ | ✓ | ✓ |
| IL-2 | | | | | | ✓ |
B
CD8+ CD69+ TEM cells
Ped Male (n=10; 6-17 yrs)
Ped Female (n=8; 11-17 yrs)
Adult Male (n=7)
Adult Female (n=6)
*
**
*
Supplemental Fig 3. CD8+ T Effector Memory Response. Scatter plots showing A+D. the increases over baseline percentages of CD8+ TEM producing MIP1β, CD107a, TNFα, IL-2, IL-17A, IFN𝛾, and Granzyme B (GzmB); B+E. CD8+ TEM observed mono-, bi-, tri-, and >3-functional populations, and; C+F. specific CD8+CD69+ TEM selected multifunctional responses following co-culture with HLA-E restricted S. Typhi infected antigen presenting targets. These MF populations were selected based on those shown in a previous S. Typhi challenge study to be associated with protection from developing disease (10,11). Bars represent medians with whiskers indicating interquartile ranges. Data divided among (A-C) male pediatric (ages 6-17; n=10), female pediatric (ages 11-17; n=8), male adult (n=7), and female adult (n=6) participants; as well as (D-F) HLA-E*01:01 (n=10), HLA-E*01:03 (n=6), and HLA-E heterozygous (n=12) participants. Scatter plot statistics were analyzed by unpaired t-test. (* p<0.05; ** p<0.01)
Response Over Baseline (%)
1 Function
3 Functions
>3 Functions
2 Functions

## Slide 6
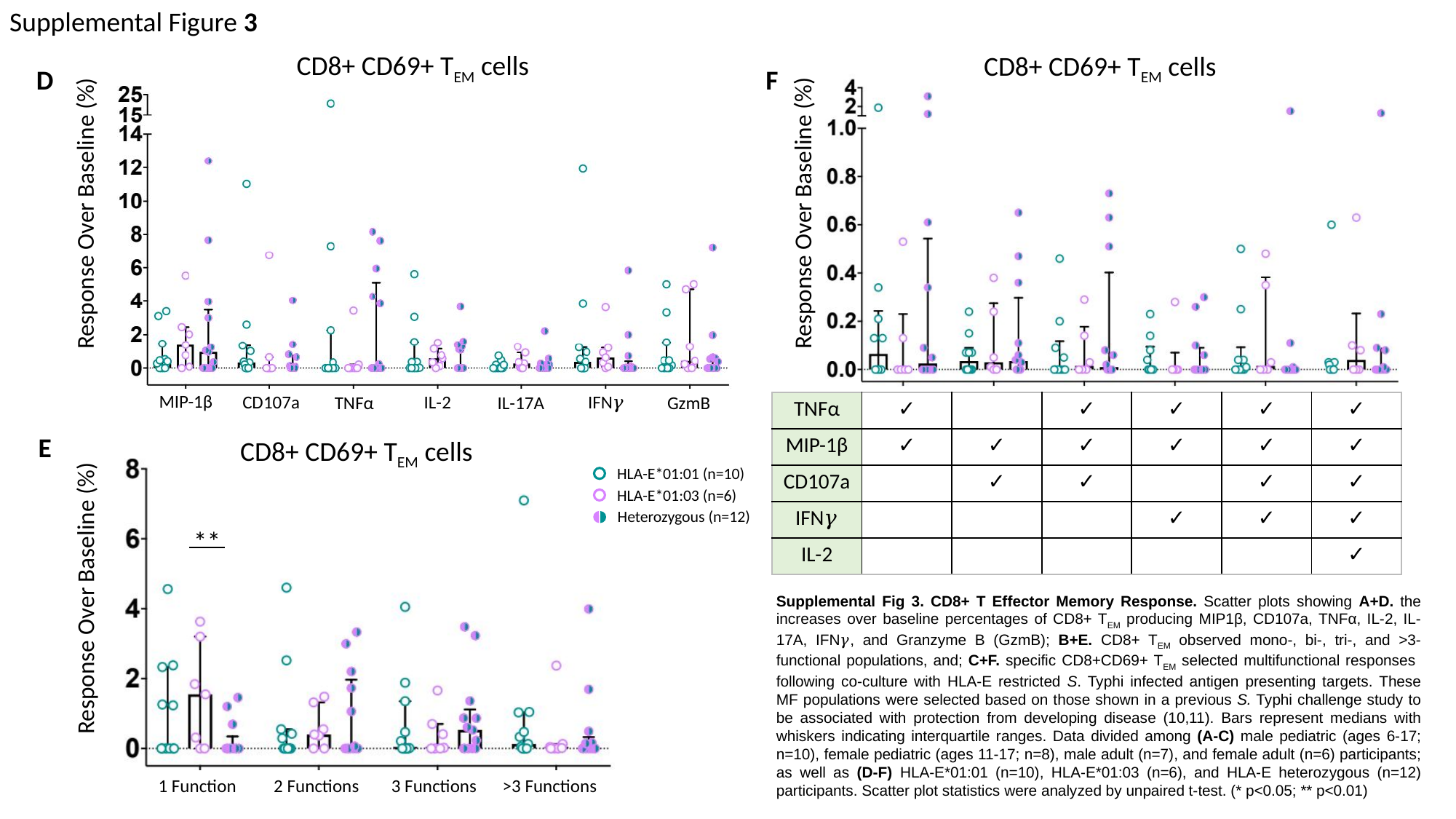

Supplemental Figure 3
CD8+ CD69+ TEM cells
CD8+ CD69+ TEM cells
F
D
Response Over Baseline (%)
Response Over Baseline (%)
MIP-1β
CD107a
IFN𝛾
IL-2
TNFα
IL-17A
GzmB
| TNFα | ✓ | | ✓ | ✓ | ✓ | ✓ |
| --- | --- | --- | --- | --- | --- | --- |
| MIP-1β | ✓ | ✓ | ✓ | ✓ | ✓ | ✓ |
| CD107a | | ✓ | ✓ | | ✓ | ✓ |
| IFN𝛾 | | | | ✓ | ✓ | ✓ |
| IL-2 | | | | | | ✓ |
E
CD8+ CD69+ TEM cells
HLA-E*01:01 (n=10)
HLA-E*01:03 (n=6)
Heterozygous (n=12)
**
Response Over Baseline (%)
Supplemental Fig 3. CD8+ T Effector Memory Response. Scatter plots showing A+D. the increases over baseline percentages of CD8+ TEM producing MIP1β, CD107a, TNFα, IL-2, IL-17A, IFN𝛾, and Granzyme B (GzmB); B+E. CD8+ TEM observed mono-, bi-, tri-, and >3-functional populations, and; C+F. specific CD8+CD69+ TEM selected multifunctional responses following co-culture with HLA-E restricted S. Typhi infected antigen presenting targets. These MF populations were selected based on those shown in a previous S. Typhi challenge study to be associated with protection from developing disease (10,11). Bars represent medians with whiskers indicating interquartile ranges. Data divided among (A-C) male pediatric (ages 6-17; n=10), female pediatric (ages 11-17; n=8), male adult (n=7), and female adult (n=6) participants; as well as (D-F) HLA-E*01:01 (n=10), HLA-E*01:03 (n=6), and HLA-E heterozygous (n=12) participants. Scatter plot statistics were analyzed by unpaired t-test. (* p<0.05; ** p<0.01)
3 Functions
2 Functions
1 Function
>3 Functions

## Slide 7
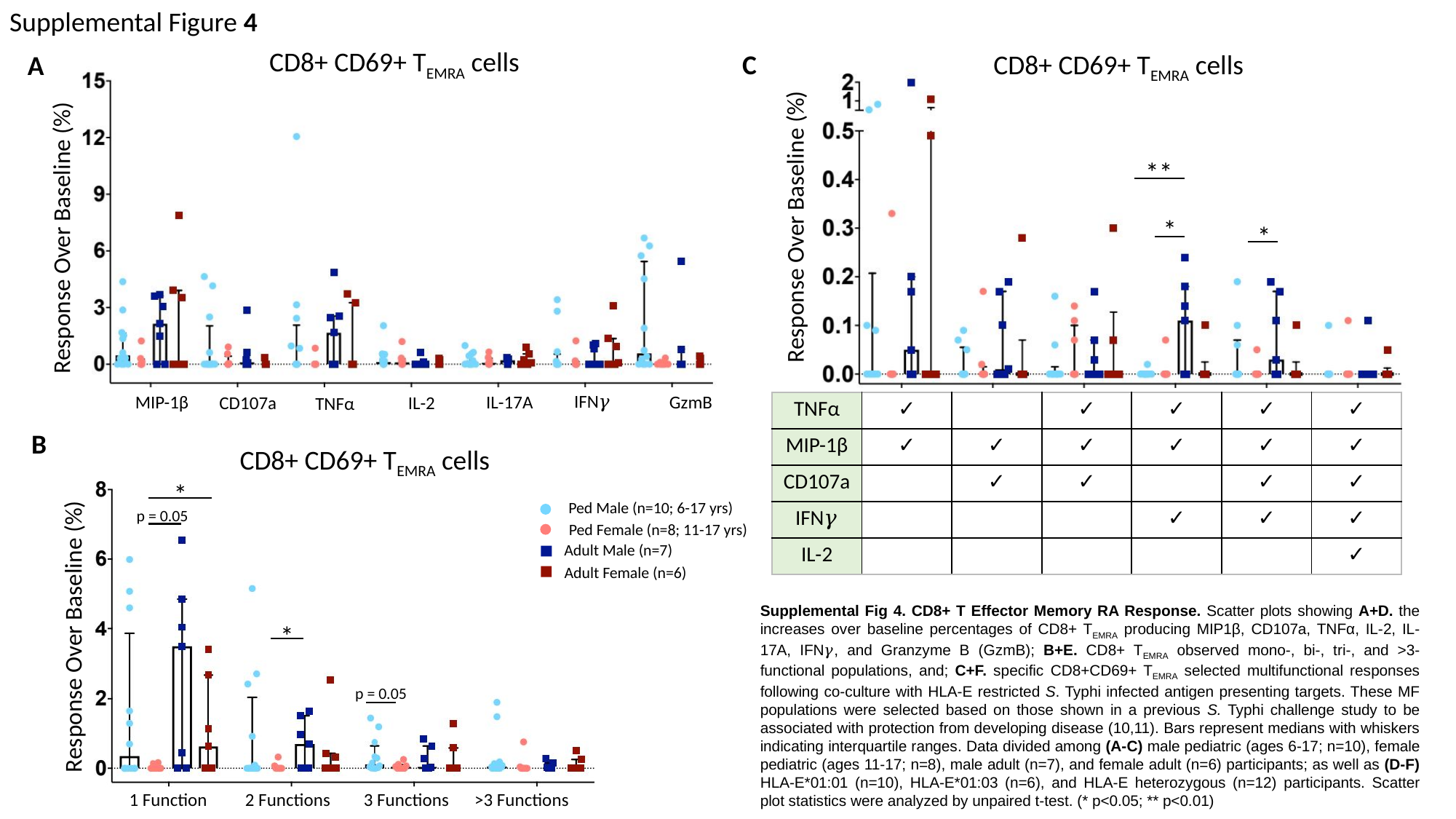

Supplemental Figure 4
CD8+ CD69+ TEMRA cells
C
CD8+ CD69+ TEMRA cells
A
**
Response Over Baseline (%)
*
*
Response Over Baseline (%)
IFN𝛾
MIP-1β
IL-17A
GzmB
CD107a
IL-2
TNFα
| TNFα | ✓ | | ✓ | ✓ | ✓ | ✓ |
| --- | --- | --- | --- | --- | --- | --- |
| MIP-1β | ✓ | ✓ | ✓ | ✓ | ✓ | ✓ |
| CD107a | | ✓ | ✓ | | ✓ | ✓ |
| IFN𝛾 | | | | ✓ | ✓ | ✓ |
| IL-2 | | | | | | ✓ |
B
CD8+ CD69+ TEMRA cells
*
Ped Male (n=10; 6-17 yrs)
Ped Female (n=8; 11-17 yrs)
Adult Male (n=7)
Adult Female (n=6)
p = 0.05
Supplemental Fig 4. CD8+ T Effector Memory RA Response. Scatter plots showing A+D. the increases over baseline percentages of CD8+ TEMRA producing MIP1β, CD107a, TNFα, IL-2, IL-17A, IFN𝛾, and Granzyme B (GzmB); B+E. CD8+ TEMRA observed mono-, bi-, tri-, and >3-functional populations, and; C+F. specific CD8+CD69+ TEMRA selected multifunctional responses following co-culture with HLA-E restricted S. Typhi infected antigen presenting targets. These MF populations were selected based on those shown in a previous S. Typhi challenge study to be associated with protection from developing disease (10,11). Bars represent medians with whiskers indicating interquartile ranges. Data divided among (A-C) male pediatric (ages 6-17; n=10), female pediatric (ages 11-17; n=8), male adult (n=7), and female adult (n=6) participants; as well as (D-F) HLA-E*01:01 (n=10), HLA-E*01:03 (n=6), and HLA-E heterozygous (n=12) participants. Scatter plot statistics were analyzed by unpaired t-test. (* p<0.05; ** p<0.01)
*
Response Over Baseline (%)
p = 0.05
>3 Functions
2 Functions
3 Functions
1 Function

## Slide 8
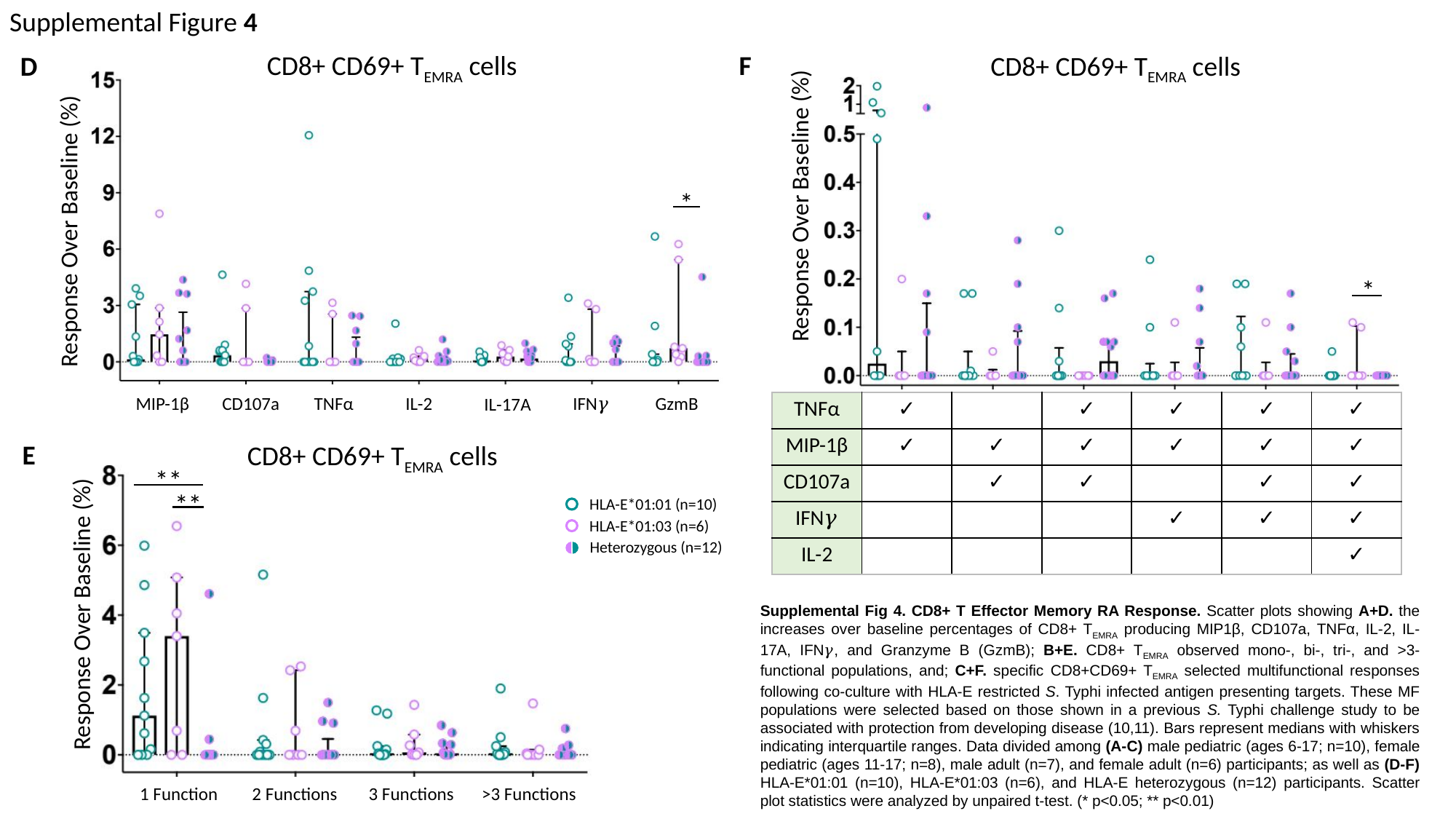

Supplemental Figure 4
CD8+ CD69+ TEMRA cells
F
D
CD8+ CD69+ TEMRA cells
*
Response Over Baseline (%)
Response Over Baseline (%)
*
GzmB
TNFα
IL-2
IFN𝛾
MIP-1β
CD107a
IL-17A
| TNFα | ✓ | | ✓ | ✓ | ✓ | ✓ |
| --- | --- | --- | --- | --- | --- | --- |
| MIP-1β | ✓ | ✓ | ✓ | ✓ | ✓ | ✓ |
| CD107a | | ✓ | ✓ | | ✓ | ✓ |
| IFN𝛾 | | | | ✓ | ✓ | ✓ |
| IL-2 | | | | | | ✓ |
E
CD8+ CD69+ TEMRA cells
**
**
HLA-E*01:01 (n=10)
HLA-E*01:03 (n=6)
Heterozygous (n=12)
Response Over Baseline (%)
Supplemental Fig 4. CD8+ T Effector Memory RA Response. Scatter plots showing A+D. the increases over baseline percentages of CD8+ TEMRA producing MIP1β, CD107a, TNFα, IL-2, IL-17A, IFN𝛾, and Granzyme B (GzmB); B+E. CD8+ TEMRA observed mono-, bi-, tri-, and >3-functional populations, and; C+F. specific CD8+CD69+ TEMRA selected multifunctional responses following co-culture with HLA-E restricted S. Typhi infected antigen presenting targets. These MF populations were selected based on those shown in a previous S. Typhi challenge study to be associated with protection from developing disease (10,11). Bars represent medians with whiskers indicating interquartile ranges. Data divided among (A-C) male pediatric (ages 6-17; n=10), female pediatric (ages 11-17; n=8), male adult (n=7), and female adult (n=6) participants; as well as (D-F) HLA-E*01:01 (n=10), HLA-E*01:03 (n=6), and HLA-E heterozygous (n=12) participants. Scatter plot statistics were analyzed by unpaired t-test. (* p<0.05; ** p<0.01)
1 Function
3 Functions
>3 Functions
2 Functions

## Slide 9
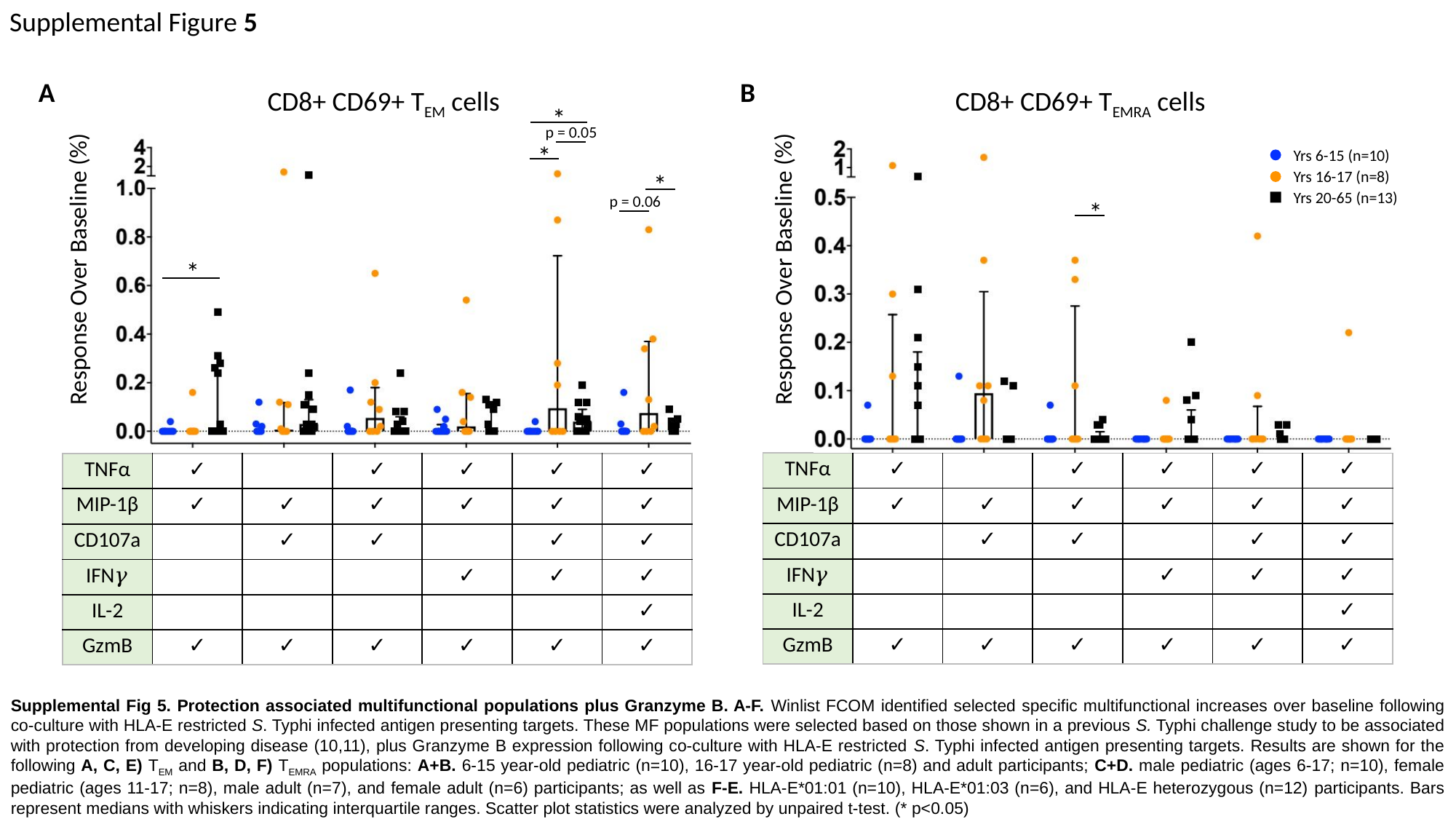

Supplemental Figure 5
A
B
CD8+ CD69+ TEM cells
CD8+ CD69+ TEMRA cells
*
p = 0.05
*
Yrs 6-15 (n=10)
Yrs 16-17 (n=8)
Yrs 20-65 (n=13)
*
p = 0.06
*
Response Over Baseline (%)
Response Over Baseline (%)
*
| TNFα | ✓ | | ✓ | ✓ | ✓ | ✓ |
| --- | --- | --- | --- | --- | --- | --- |
| MIP-1β | ✓ | ✓ | ✓ | ✓ | ✓ | ✓ |
| CD107a | | ✓ | ✓ | | ✓ | ✓ |
| IFN𝛾 | | | | ✓ | ✓ | ✓ |
| IL-2 | | | | | | ✓ |
| GzmB | ✓ | ✓ | ✓ | ✓ | ✓ | ✓ |
| TNFα | ✓ | | ✓ | ✓ | ✓ | ✓ |
| --- | --- | --- | --- | --- | --- | --- |
| MIP-1β | ✓ | ✓ | ✓ | ✓ | ✓ | ✓ |
| CD107a | | ✓ | ✓ | | ✓ | ✓ |
| IFN𝛾 | | | | ✓ | ✓ | ✓ |
| IL-2 | | | | | | ✓ |
| GzmB | ✓ | ✓ | ✓ | ✓ | ✓ | ✓ |
Supplemental Fig 5. Protection associated multifunctional populations plus Granzyme B. A-F. Winlist FCOM identified selected specific multifunctional increases over baseline following co-culture with HLA-E restricted S. Typhi infected antigen presenting targets. These MF populations were selected based on those shown in a previous S. Typhi challenge study to be associated with protection from developing disease (10,11), plus Granzyme B expression following co-culture with HLA-E restricted S. Typhi infected antigen presenting targets. Results are shown for the following A, C, E) TEM and B, D, F) TEMRA populations: A+B. 6-15 year-old pediatric (n=10), 16-17 year-old pediatric (n=8) and adult participants; C+D. male pediatric (ages 6-17; n=10), female pediatric (ages 11-17; n=8), male adult (n=7), and female adult (n=6) participants; as well as F-E. HLA-E*01:01 (n=10), HLA-E*01:03 (n=6), and HLA-E heterozygous (n=12) participants. Bars represent medians with whiskers indicating interquartile ranges. Scatter plot statistics were analyzed by unpaired t-test. (* p<0.05)

## Slide 10
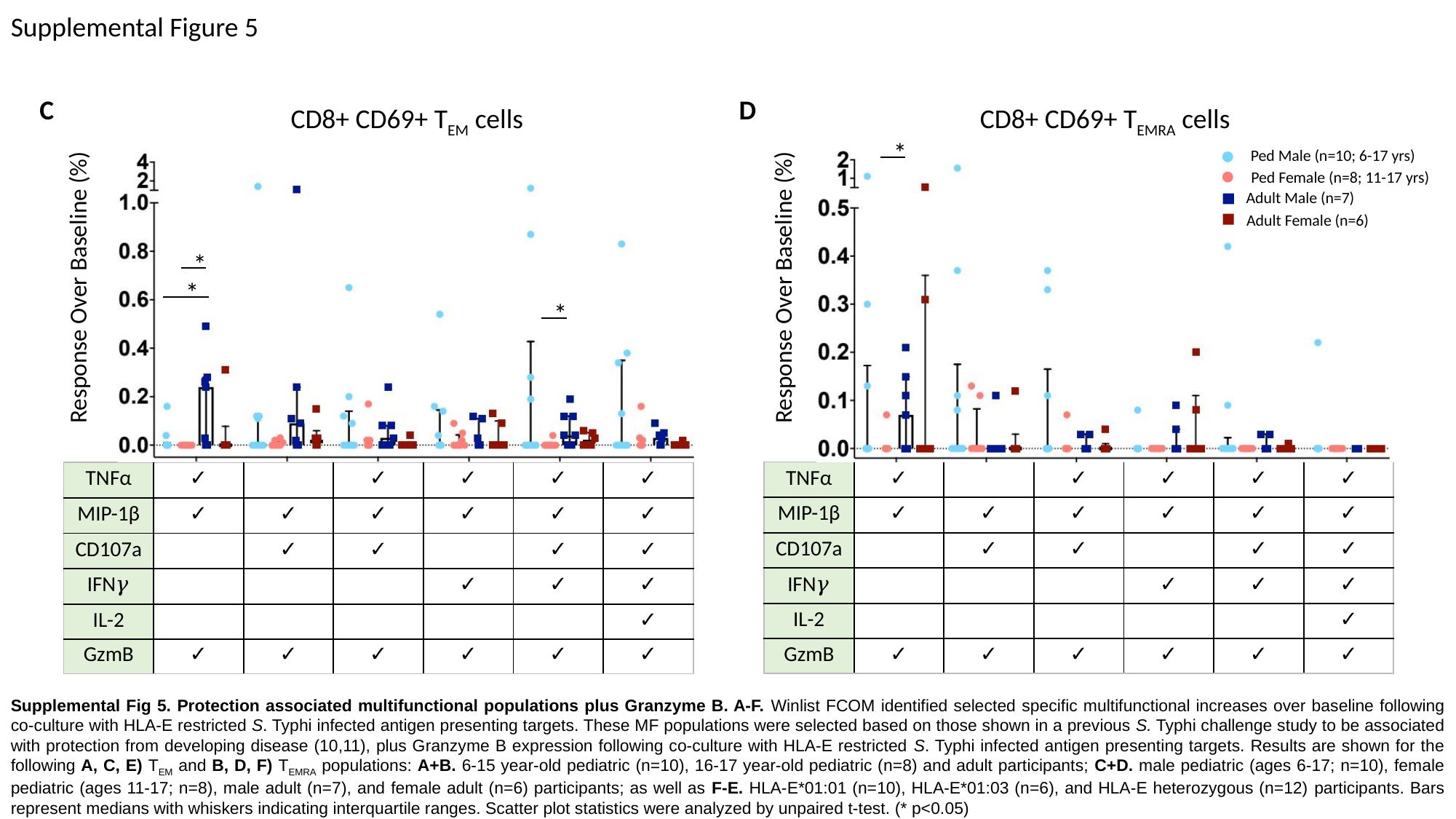

Supplemental Figure 5
C
D
CD8+ CD69+ TEM cells
CD8+ CD69+ TEMRA cells
*
Ped Male (n=10; 6-17 yrs)
Ped Female (n=8; 11-17 yrs)
Adult Male (n=7)
Adult Female (n=6)
*
Response Over Baseline (%)
Response Over Baseline (%)
*
*
| TNFα | ✓ | | ✓ | ✓ | ✓ | ✓ |
| --- | --- | --- | --- | --- | --- | --- |
| MIP-1β | ✓ | ✓ | ✓ | ✓ | ✓ | ✓ |
| CD107a | | ✓ | ✓ | | ✓ | ✓ |
| IFN𝛾 | | | | ✓ | ✓ | ✓ |
| IL-2 | | | | | | ✓ |
| GzmB | ✓ | ✓ | ✓ | ✓ | ✓ | ✓ |
| TNFα | ✓ | | ✓ | ✓ | ✓ | ✓ |
| --- | --- | --- | --- | --- | --- | --- |
| MIP-1β | ✓ | ✓ | ✓ | ✓ | ✓ | ✓ |
| CD107a | | ✓ | ✓ | | ✓ | ✓ |
| IFN𝛾 | | | | ✓ | ✓ | ✓ |
| IL-2 | | | | | | ✓ |
| GzmB | ✓ | ✓ | ✓ | ✓ | ✓ | ✓ |
Supplemental Fig 5. Protection associated multifunctional populations plus Granzyme B. A-F. Winlist FCOM identified selected specific multifunctional increases over baseline following co-culture with HLA-E restricted S. Typhi infected antigen presenting targets. These MF populations were selected based on those shown in a previous S. Typhi challenge study to be associated with protection from developing disease (10,11), plus Granzyme B expression following co-culture with HLA-E restricted S. Typhi infected antigen presenting targets. Results are shown for the following A, C, E) TEM and B, D, F) TEMRA populations: A+B. 6-15 year-old pediatric (n=10), 16-17 year-old pediatric (n=8) and adult participants; C+D. male pediatric (ages 6-17; n=10), female pediatric (ages 11-17; n=8), male adult (n=7), and female adult (n=6) participants; as well as F-E. HLA-E*01:01 (n=10), HLA-E*01:03 (n=6), and HLA-E heterozygous (n=12) participants. Bars represent medians with whiskers indicating interquartile ranges. Scatter plot statistics were analyzed by unpaired t-test. (* p<0.05)

## Slide 11
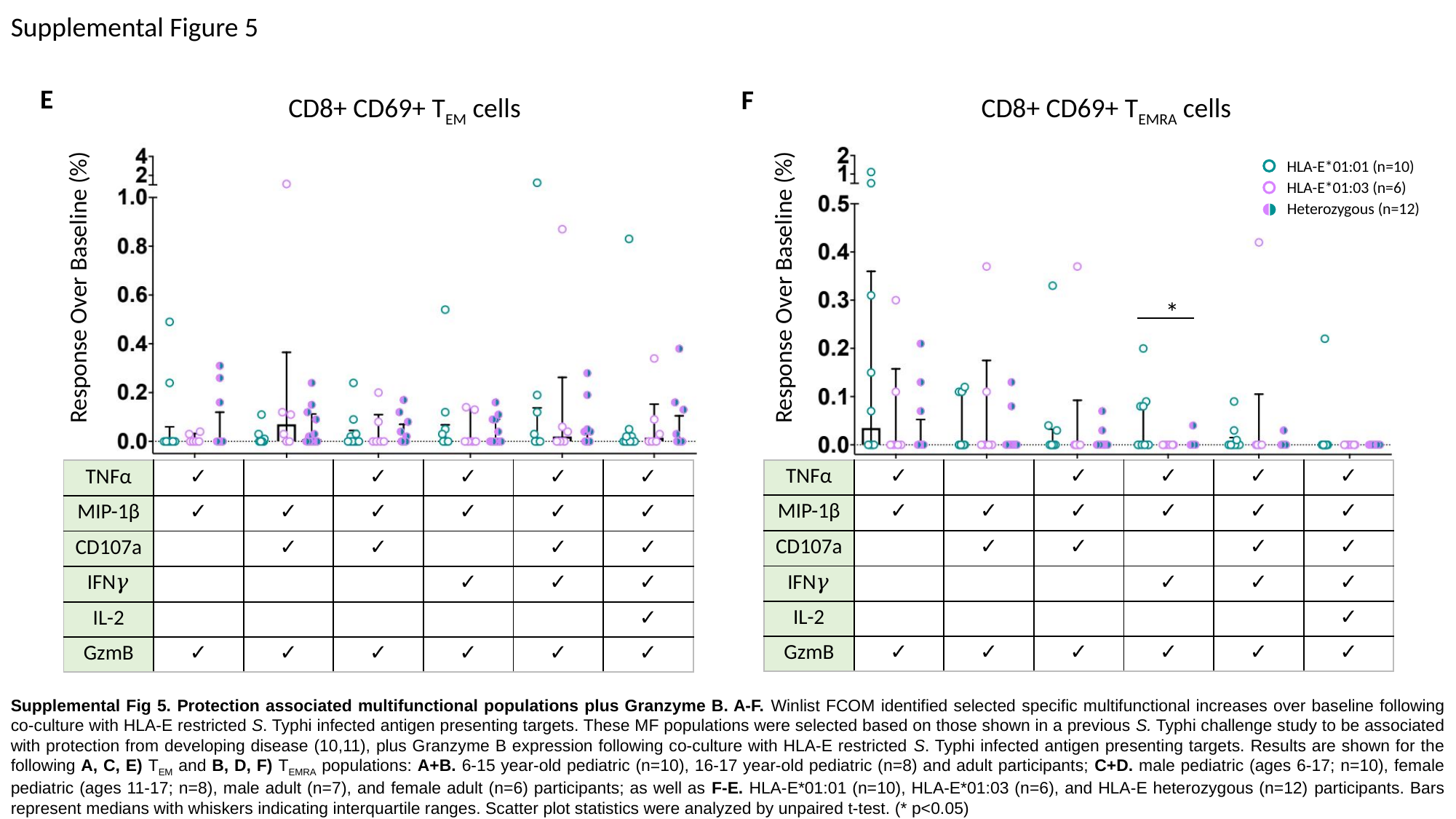

Supplemental Figure 5
E
F
CD8+ CD69+ TEM cells
CD8+ CD69+ TEMRA cells
HLA-E*01:01 (n=10)
HLA-E*01:03 (n=6)
Heterozygous (n=12)
Response Over Baseline (%)
Response Over Baseline (%)
*
| TNFα | ✓ | | ✓ | ✓ | ✓ | ✓ |
| --- | --- | --- | --- | --- | --- | --- |
| MIP-1β | ✓ | ✓ | ✓ | ✓ | ✓ | ✓ |
| CD107a | | ✓ | ✓ | | ✓ | ✓ |
| IFN𝛾 | | | | ✓ | ✓ | ✓ |
| IL-2 | | | | | | ✓ |
| GzmB | ✓ | ✓ | ✓ | ✓ | ✓ | ✓ |
| TNFα | ✓ | | ✓ | ✓ | ✓ | ✓ |
| --- | --- | --- | --- | --- | --- | --- |
| MIP-1β | ✓ | ✓ | ✓ | ✓ | ✓ | ✓ |
| CD107a | | ✓ | ✓ | | ✓ | ✓ |
| IFN𝛾 | | | | ✓ | ✓ | ✓ |
| IL-2 | | | | | | ✓ |
| GzmB | ✓ | ✓ | ✓ | ✓ | ✓ | ✓ |
Supplemental Fig 5. Protection associated multifunctional populations plus Granzyme B. A-F. Winlist FCOM identified selected specific multifunctional increases over baseline following co-culture with HLA-E restricted S. Typhi infected antigen presenting targets. These MF populations were selected based on those shown in a previous S. Typhi challenge study to be associated with protection from developing disease (10,11), plus Granzyme B expression following co-culture with HLA-E restricted S. Typhi infected antigen presenting targets. Results are shown for the following A, C, E) TEM and B, D, F) TEMRA populations: A+B. 6-15 year-old pediatric (n=10), 16-17 year-old pediatric (n=8) and adult participants; C+D. male pediatric (ages 6-17; n=10), female pediatric (ages 11-17; n=8), male adult (n=7), and female adult (n=6) participants; as well as F-E. HLA-E*01:01 (n=10), HLA-E*01:03 (n=6), and HLA-E heterozygous (n=12) participants. Bars represent medians with whiskers indicating interquartile ranges. Scatter plot statistics were analyzed by unpaired t-test. (* p<0.05)

## Slide 12
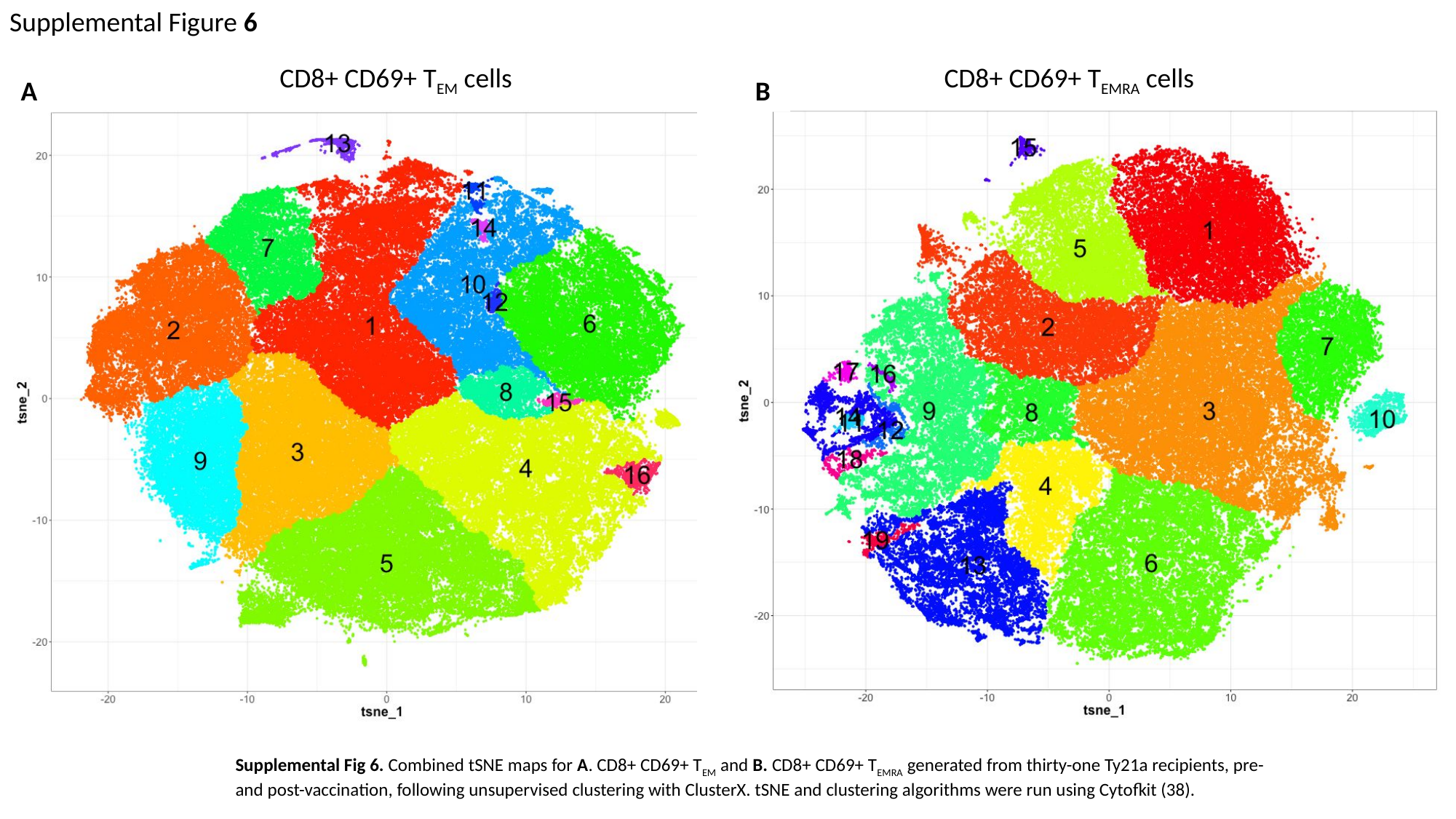

Supplemental Figure 6
CD8+ CD69+ TEM cells
CD8+ CD69+ TEMRA cells
A
B
Supplemental Fig 6. Combined tSNE maps for A. CD8+ CD69+ TEM and B. CD8+ CD69+ TEMRA generated from thirty-one Ty21a recipients, pre- and post-vaccination, following unsupervised clustering with ClusterX. tSNE and clustering algorithms were run using Cytofkit (38).

## Slide 13
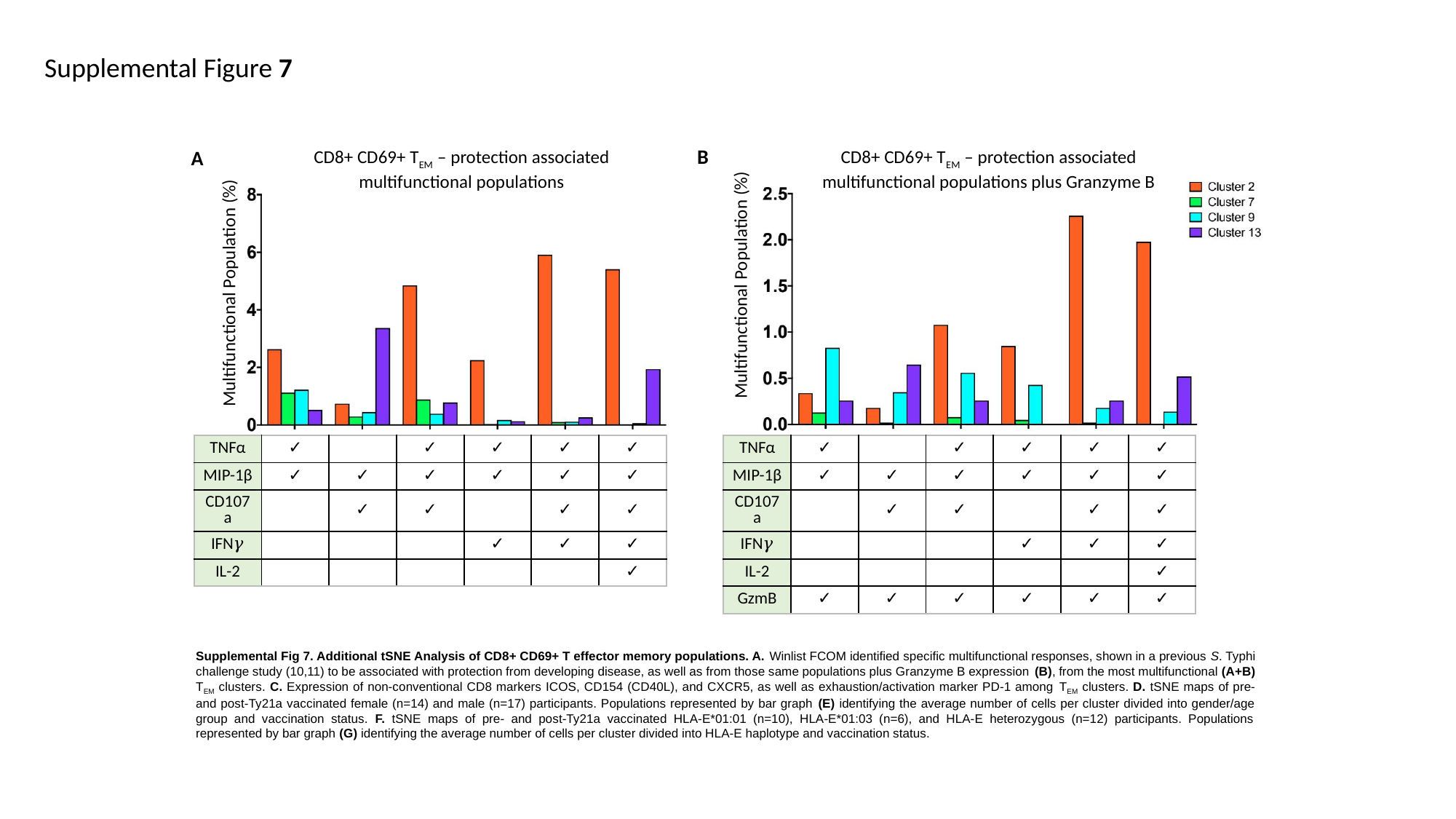

Supplemental Figure 7
B
A
CD8+ CD69+ TEM – protection associated multifunctional populations
CD8+ CD69+ TEM – protection associated multifunctional populations plus Granzyme B
Multifunctional Population (%)
Multifunctional Population (%)
| TNFα | ✓ | | ✓ | ✓ | ✓ | ✓ |
| --- | --- | --- | --- | --- | --- | --- |
| MIP-1β | ✓ | ✓ | ✓ | ✓ | ✓ | ✓ |
| CD107a | | ✓ | ✓ | | ✓ | ✓ |
| IFN𝛾 | | | | ✓ | ✓ | ✓ |
| IL-2 | | | | | | ✓ |
| TNFα | ✓ | | ✓ | ✓ | ✓ | ✓ |
| --- | --- | --- | --- | --- | --- | --- |
| MIP-1β | ✓ | ✓ | ✓ | ✓ | ✓ | ✓ |
| CD107a | | ✓ | ✓ | | ✓ | ✓ |
| IFN𝛾 | | | | ✓ | ✓ | ✓ |
| IL-2 | | | | | | ✓ |
| GzmB | ✓ | ✓ | ✓ | ✓ | ✓ | ✓ |
Supplemental Fig 7. Additional tSNE Analysis of CD8+ CD69+ T effector memory populations. A. Winlist FCOM identified specific multifunctional responses, shown in a previous S. Typhi challenge study (10,11) to be associated with protection from developing disease, as well as from those same populations plus Granzyme B expression (B), from the most multifunctional (A+B) TEM clusters. C. Expression of non-conventional CD8 markers ICOS, CD154 (CD40L), and CXCR5, as well as exhaustion/activation marker PD-1 among TEM clusters. D. tSNE maps of pre- and post-Ty21a vaccinated female (n=14) and male (n=17) participants. Populations represented by bar graph (E) identifying the average number of cells per cluster divided into gender/age group and vaccination status. F. tSNE maps of pre- and post-Ty21a vaccinated HLA-E*01:01 (n=10), HLA-E*01:03 (n=6), and HLA-E heterozygous (n=12) participants. Populations represented by bar graph (G) identifying the average number of cells per cluster divided into HLA-E haplotype and vaccination status.

## Slide 14
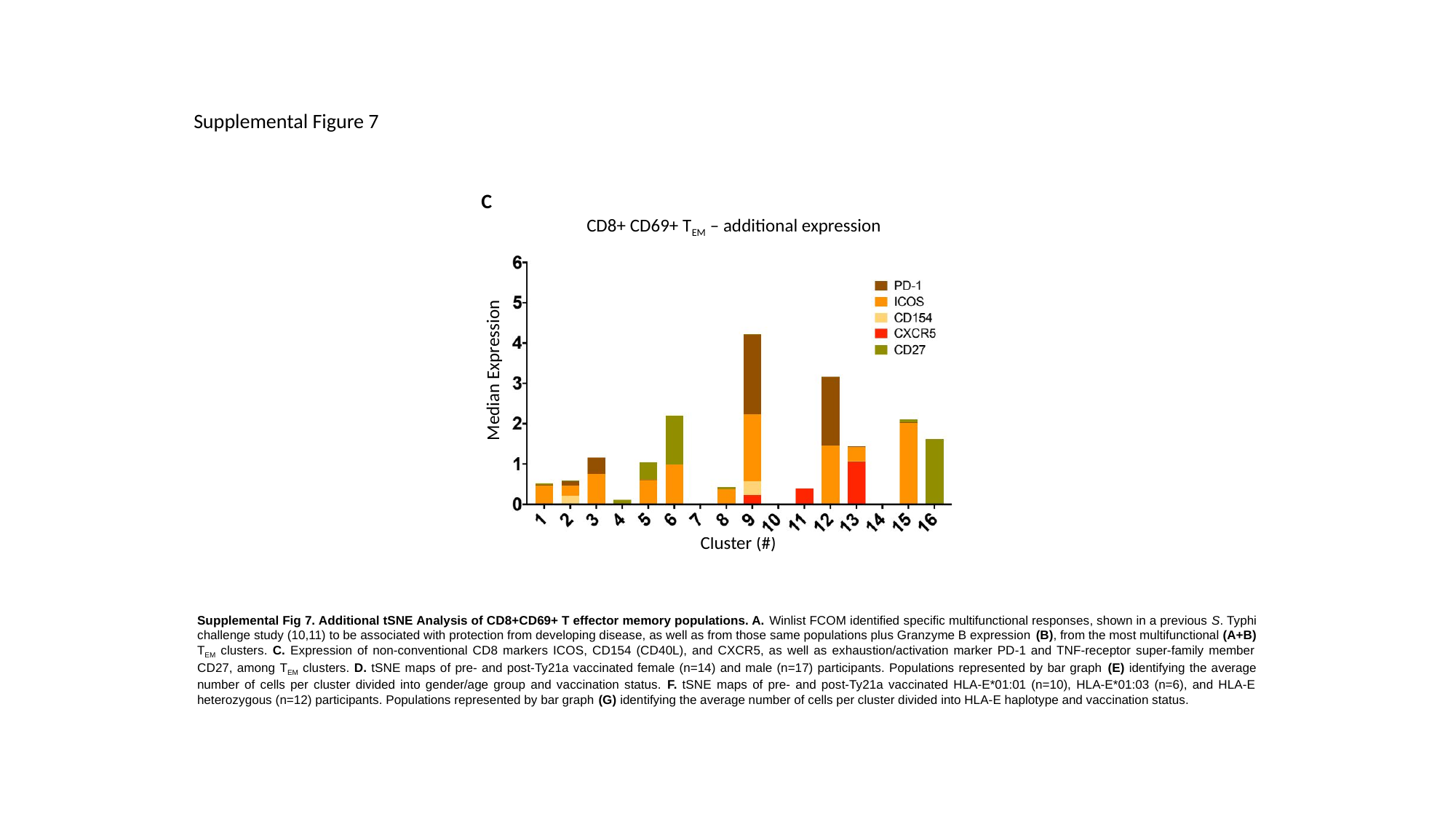

Supplemental Figure 7
C
CD8+ CD69+ TEM – additional expression
Median Expression
Cluster (#)
Supplemental Fig 7. Additional tSNE Analysis of CD8+CD69+ T effector memory populations. A. Winlist FCOM identified specific multifunctional responses, shown in a previous S. Typhi challenge study (10,11) to be associated with protection from developing disease, as well as from those same populations plus Granzyme B expression (B), from the most multifunctional (A+B) TEM clusters. C. Expression of non-conventional CD8 markers ICOS, CD154 (CD40L), and CXCR5, as well as exhaustion/activation marker PD-1 and TNF-receptor super-family member CD27, among TEM clusters. D. tSNE maps of pre- and post-Ty21a vaccinated female (n=14) and male (n=17) participants. Populations represented by bar graph (E) identifying the average number of cells per cluster divided into gender/age group and vaccination status. F. tSNE maps of pre- and post-Ty21a vaccinated HLA-E*01:01 (n=10), HLA-E*01:03 (n=6), and HLA-E heterozygous (n=12) participants. Populations represented by bar graph (G) identifying the average number of cells per cluster divided into HLA-E haplotype and vaccination status.

## Slide 15
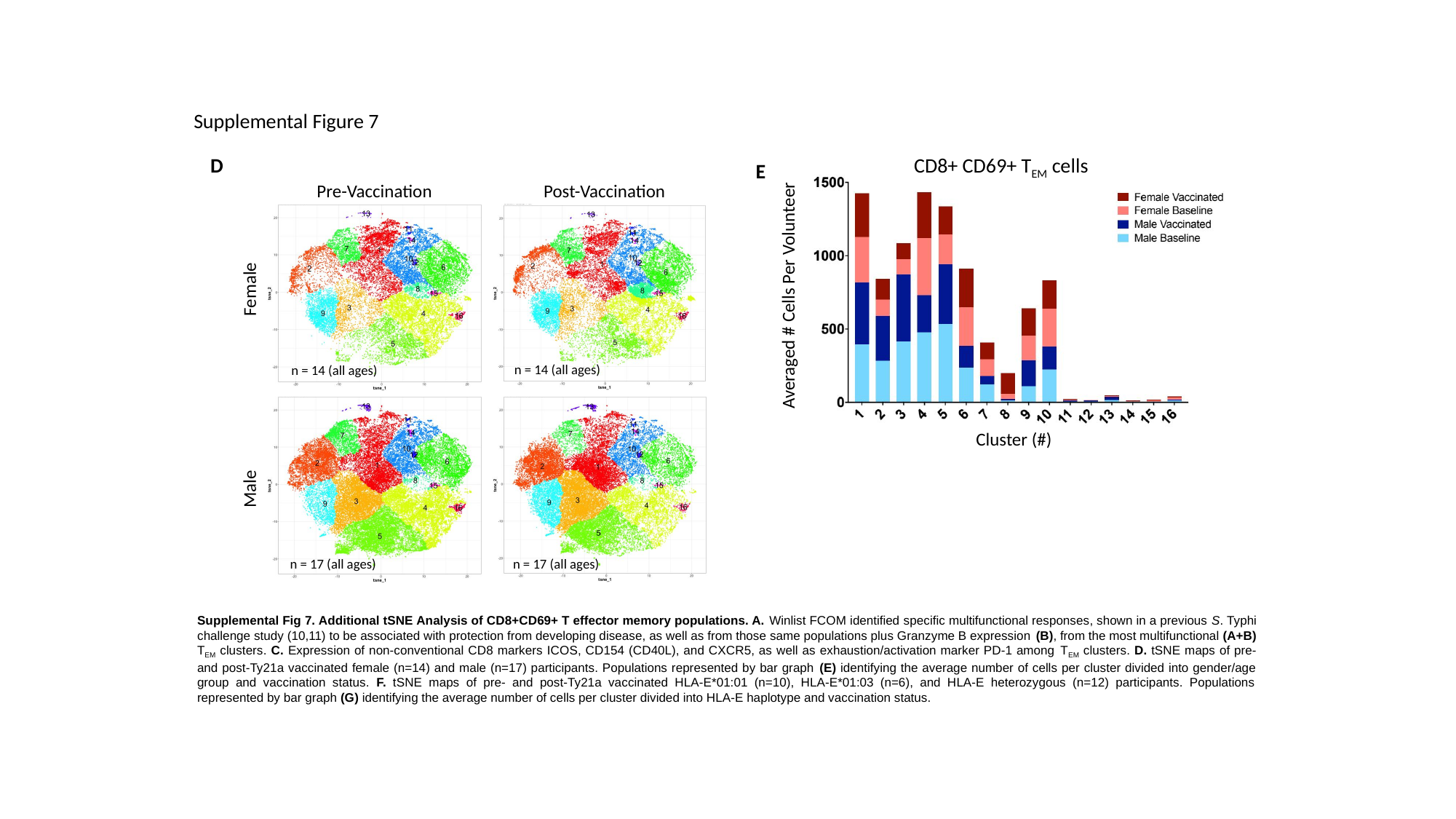

Supplemental Figure 7
CD8+ CD69+ TEM cells
D
E
Post-Vaccination
Pre-Vaccination
Female
Averaged # Cells Per Volunteer
n = 14 (all ages)
n = 14 (all ages)
Cluster (#)
Male
n = 17 (all ages)
n = 17 (all ages)
Supplemental Fig 7. Additional tSNE Analysis of CD8+CD69+ T effector memory populations. A. Winlist FCOM identified specific multifunctional responses, shown in a previous S. Typhi challenge study (10,11) to be associated with protection from developing disease, as well as from those same populations plus Granzyme B expression (B), from the most multifunctional (A+B) TEM clusters. C. Expression of non-conventional CD8 markers ICOS, CD154 (CD40L), and CXCR5, as well as exhaustion/activation marker PD-1 among TEM clusters. D. tSNE maps of pre- and post-Ty21a vaccinated female (n=14) and male (n=17) participants. Populations represented by bar graph (E) identifying the average number of cells per cluster divided into gender/age group and vaccination status. F. tSNE maps of pre- and post-Ty21a vaccinated HLA-E*01:01 (n=10), HLA-E*01:03 (n=6), and HLA-E heterozygous (n=12) participants. Populations represented by bar graph (G) identifying the average number of cells per cluster divided into HLA-E haplotype and vaccination status.

## Slide 16
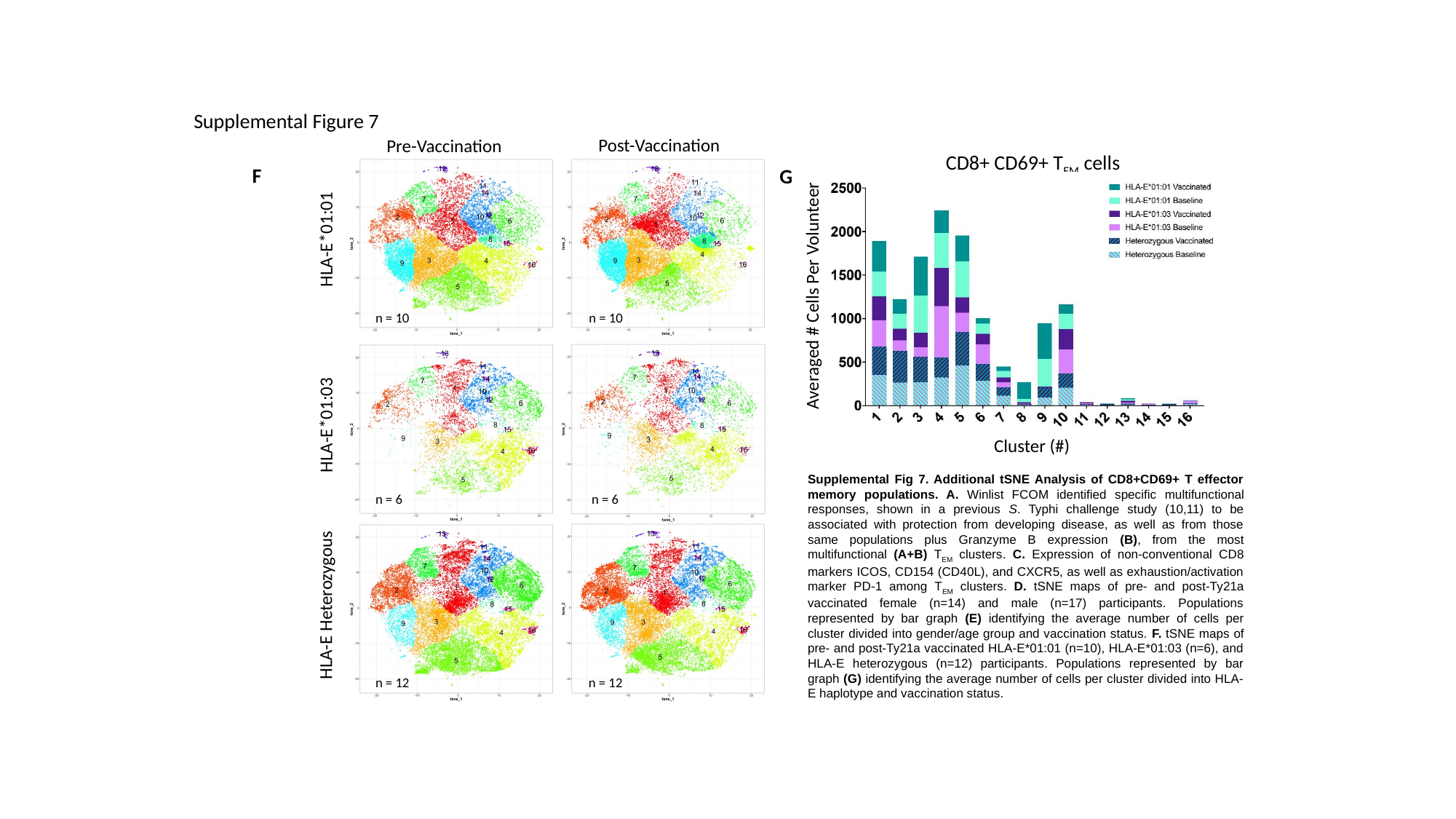

Supplemental Figure 7
Post-Vaccination
Pre-Vaccination
CD8+ CD69+ TEM cells
F
G
HLA-E*01:01
Averaged # Cells Per Volunteer
n = 10
n = 10
HLA-E*01:03
Cluster (#)
Supplemental Fig 7. Additional tSNE Analysis of CD8+CD69+ T effector memory populations. A. Winlist FCOM identified specific multifunctional responses, shown in a previous S. Typhi challenge study (10,11) to be associated with protection from developing disease, as well as from those same populations plus Granzyme B expression (B), from the most multifunctional (A+B) TEM clusters. C. Expression of non-conventional CD8 markers ICOS, CD154 (CD40L), and CXCR5, as well as exhaustion/activation marker PD-1 among TEM clusters. D. tSNE maps of pre- and post-Ty21a vaccinated female (n=14) and male (n=17) participants. Populations represented by bar graph (E) identifying the average number of cells per cluster divided into gender/age group and vaccination status. F. tSNE maps of pre- and post-Ty21a vaccinated HLA-E*01:01 (n=10), HLA-E*01:03 (n=6), and HLA-E heterozygous (n=12) participants. Populations represented by bar graph (G) identifying the average number of cells per cluster divided into HLA-E haplotype and vaccination status.
n = 6
n = 6
HLA-E Heterozygous
n = 12
n = 12

## Slide 17
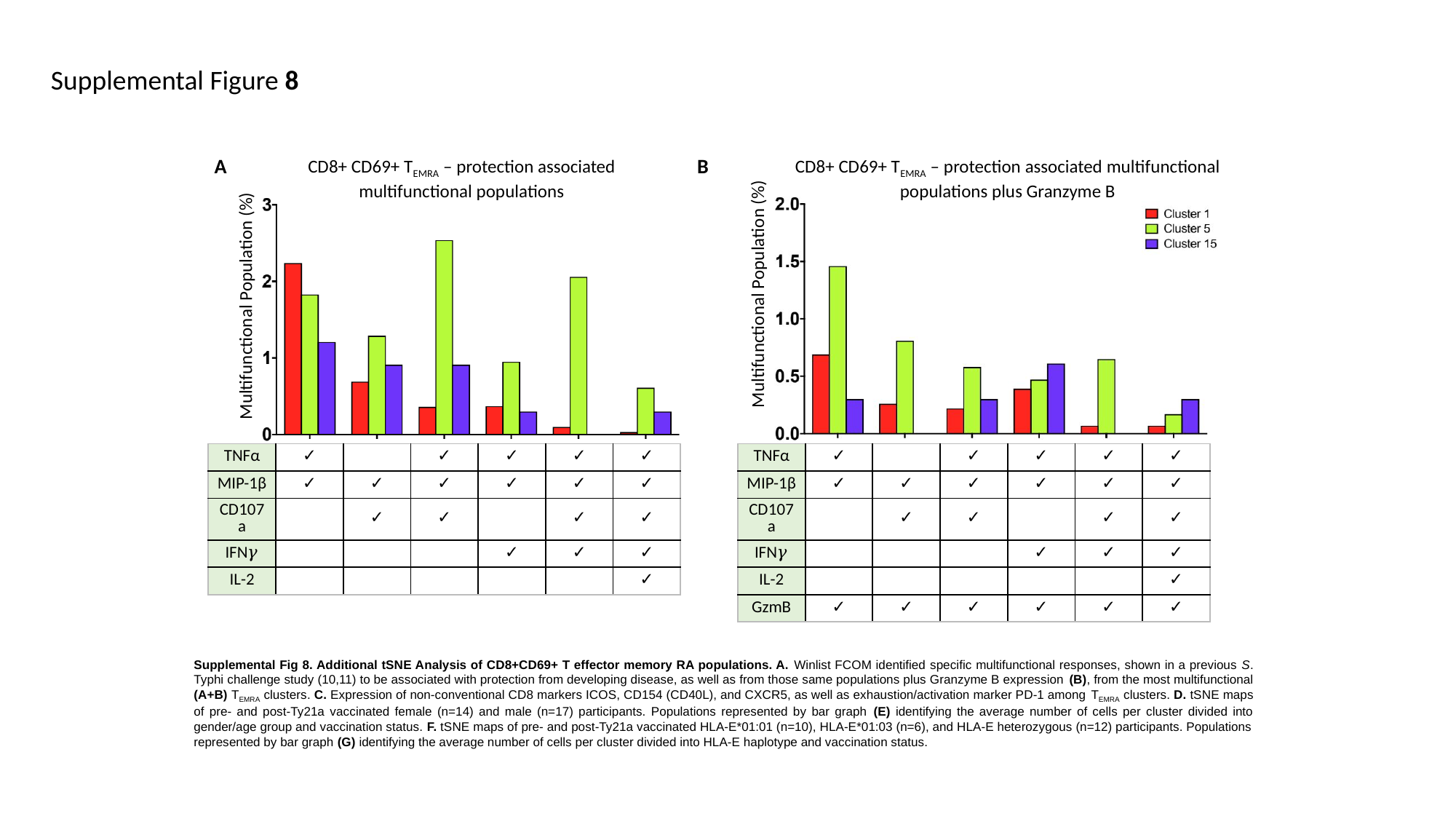

Supplemental Figure 8
A
B
CD8+ CD69+ TEMRA – protection associated multifunctional populations
CD8+ CD69+ TEMRA – protection associated multifunctional populations plus Granzyme B
Multifunctional Population (%)
Multifunctional Population (%)
| TNFα | ✓ | | ✓ | ✓ | ✓ | ✓ |
| --- | --- | --- | --- | --- | --- | --- |
| MIP-1β | ✓ | ✓ | ✓ | ✓ | ✓ | ✓ |
| CD107a | | ✓ | ✓ | | ✓ | ✓ |
| IFN𝛾 | | | | ✓ | ✓ | ✓ |
| IL-2 | | | | | | ✓ |
| TNFα | ✓ | | ✓ | ✓ | ✓ | ✓ |
| --- | --- | --- | --- | --- | --- | --- |
| MIP-1β | ✓ | ✓ | ✓ | ✓ | ✓ | ✓ |
| CD107a | | ✓ | ✓ | | ✓ | ✓ |
| IFN𝛾 | | | | ✓ | ✓ | ✓ |
| IL-2 | | | | | | ✓ |
| GzmB | ✓ | ✓ | ✓ | ✓ | ✓ | ✓ |
Supplemental Fig 8. Additional tSNE Analysis of CD8+CD69+ T effector memory RA populations. A. Winlist FCOM identified specific multifunctional responses, shown in a previous S. Typhi challenge study (10,11) to be associated with protection from developing disease, as well as from those same populations plus Granzyme B expression (B), from the most multifunctional (A+B) TEMRA clusters. C. Expression of non-conventional CD8 markers ICOS, CD154 (CD40L), and CXCR5, as well as exhaustion/activation marker PD-1 among TEMRA clusters. D. tSNE maps of pre- and post-Ty21a vaccinated female (n=14) and male (n=17) participants. Populations represented by bar graph (E) identifying the average number of cells per cluster divided into gender/age group and vaccination status. F. tSNE maps of pre- and post-Ty21a vaccinated HLA-E*01:01 (n=10), HLA-E*01:03 (n=6), and HLA-E heterozygous (n=12) participants. Populations represented by bar graph (G) identifying the average number of cells per cluster divided into HLA-E haplotype and vaccination status.

## Slide 18
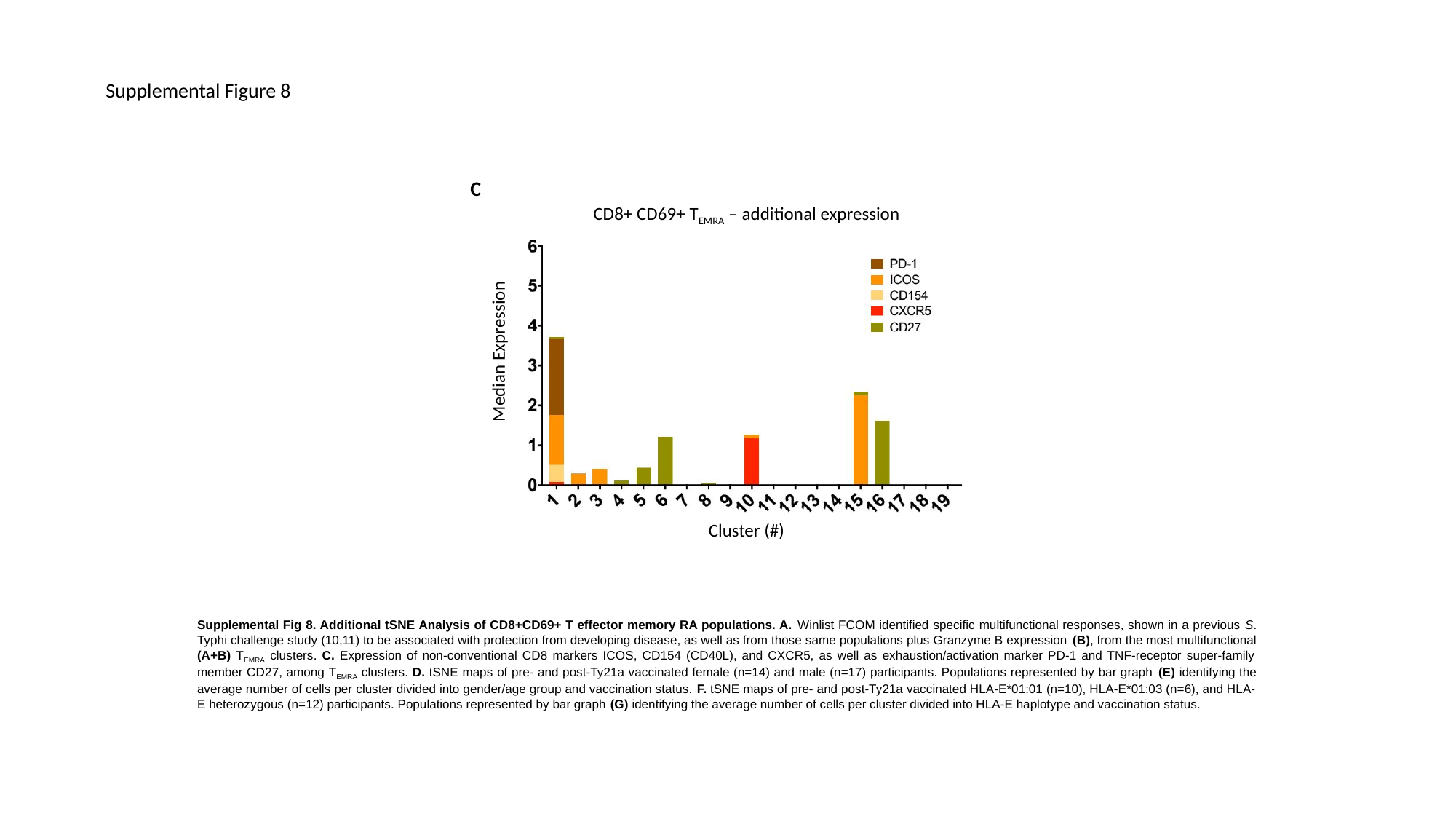

Supplemental Figure 8
C
CD8+ CD69+ TEMRA – additional expression
Median Expression
Cluster (#)
Supplemental Fig 8. Additional tSNE Analysis of CD8+CD69+ T effector memory RA populations. A. Winlist FCOM identified specific multifunctional responses, shown in a previous S. Typhi challenge study (10,11) to be associated with protection from developing disease, as well as from those same populations plus Granzyme B expression (B), from the most multifunctional (A+B) TEMRA clusters. C. Expression of non-conventional CD8 markers ICOS, CD154 (CD40L), and CXCR5, as well as exhaustion/activation marker PD-1 and TNF-receptor super-family member CD27, among TEMRA clusters. D. tSNE maps of pre- and post-Ty21a vaccinated female (n=14) and male (n=17) participants. Populations represented by bar graph (E) identifying the average number of cells per cluster divided into gender/age group and vaccination status. F. tSNE maps of pre- and post-Ty21a vaccinated HLA-E*01:01 (n=10), HLA-E*01:03 (n=6), and HLA-E heterozygous (n=12) participants. Populations represented by bar graph (G) identifying the average number of cells per cluster divided into HLA-E haplotype and vaccination status.

## Slide 19
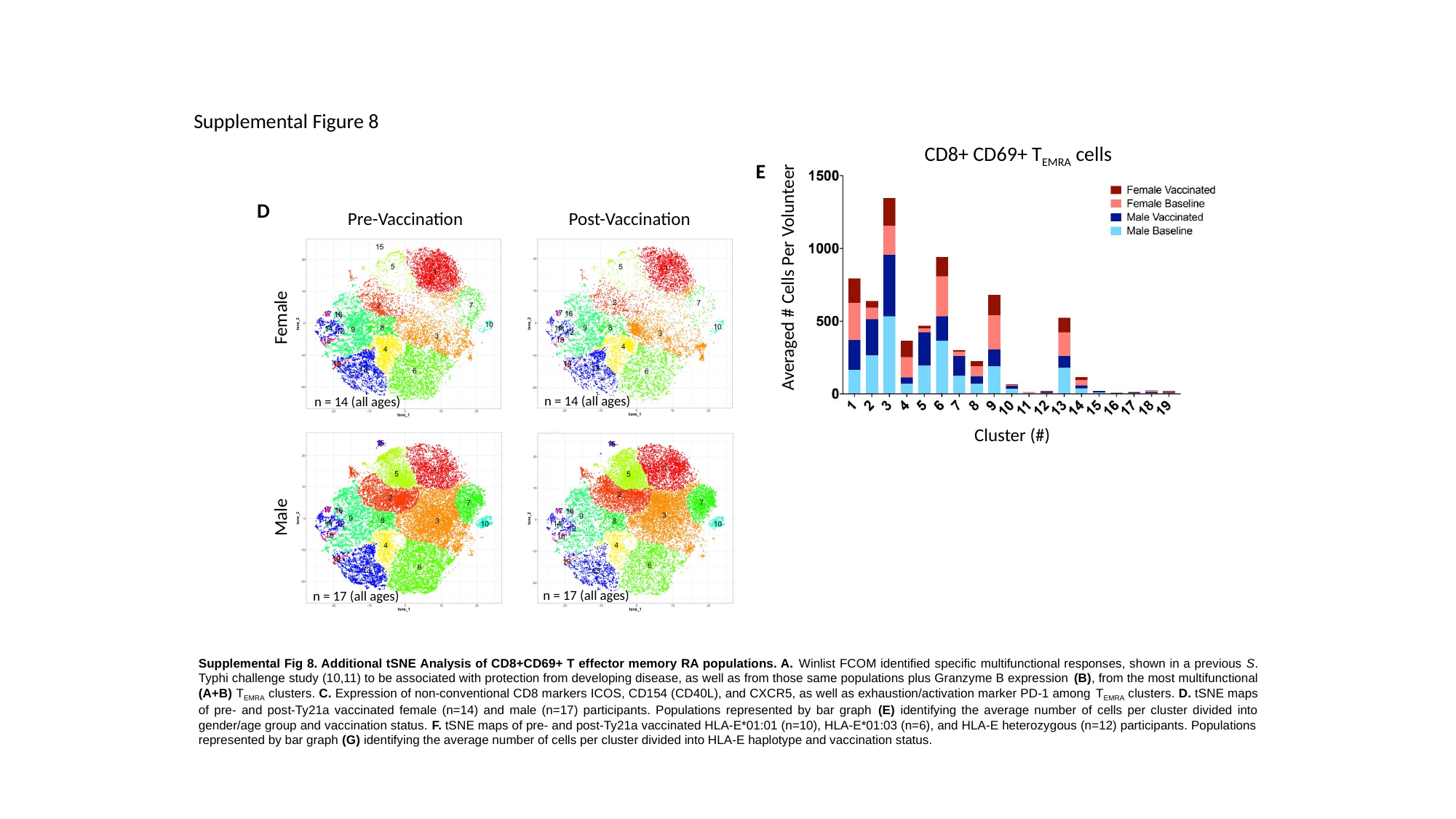

Supplemental Figure 8
CD8+ CD69+ TEMRA cells
E
D
Post-Vaccination
Pre-Vaccination
Averaged # Cells Per Volunteer
Female
n = 14 (all ages)
n = 14 (all ages)
Cluster (#)
Male
n = 17 (all ages)
n = 17 (all ages)
Supplemental Fig 8. Additional tSNE Analysis of CD8+CD69+ T effector memory RA populations. A. Winlist FCOM identified specific multifunctional responses, shown in a previous S. Typhi challenge study (10,11) to be associated with protection from developing disease, as well as from those same populations plus Granzyme B expression (B), from the most multifunctional (A+B) TEMRA clusters. C. Expression of non-conventional CD8 markers ICOS, CD154 (CD40L), and CXCR5, as well as exhaustion/activation marker PD-1 among TEMRA clusters. D. tSNE maps of pre- and post-Ty21a vaccinated female (n=14) and male (n=17) participants. Populations represented by bar graph (E) identifying the average number of cells per cluster divided into gender/age group and vaccination status. F. tSNE maps of pre- and post-Ty21a vaccinated HLA-E*01:01 (n=10), HLA-E*01:03 (n=6), and HLA-E heterozygous (n=12) participants. Populations represented by bar graph (G) identifying the average number of cells per cluster divided into HLA-E haplotype and vaccination status.

## Slide 20
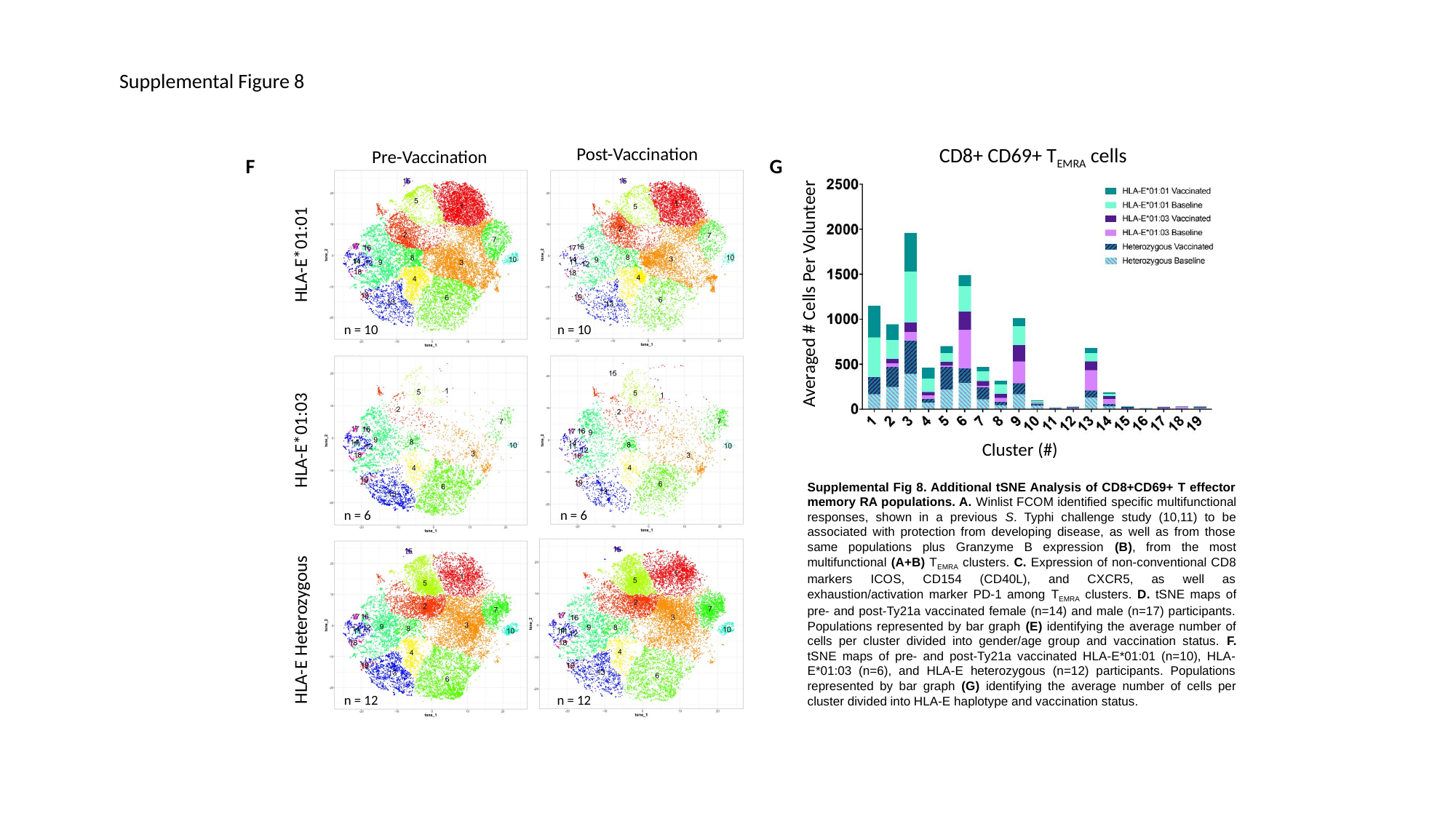

Supplemental Figure 8
CD8+ CD69+ TEMRA cells
Post-Vaccination
Pre-Vaccination
F
G
HLA-E*01:01
Averaged # Cells Per Volunteer
n = 10
n = 10
HLA-E*01:03
Cluster (#)
Supplemental Fig 8. Additional tSNE Analysis of CD8+CD69+ T effector memory RA populations. A. Winlist FCOM identified specific multifunctional responses, shown in a previous S. Typhi challenge study (10,11) to be associated with protection from developing disease, as well as from those same populations plus Granzyme B expression (B), from the most multifunctional (A+B) TEMRA clusters. C. Expression of non-conventional CD8 markers ICOS, CD154 (CD40L), and CXCR5, as well as exhaustion/activation marker PD-1 among TEMRA clusters. D. tSNE maps of pre- and post-Ty21a vaccinated female (n=14) and male (n=17) participants. Populations represented by bar graph (E) identifying the average number of cells per cluster divided into gender/age group and vaccination status. F. tSNE maps of pre- and post-Ty21a vaccinated HLA-E*01:01 (n=10), HLA-E*01:03 (n=6), and HLA-E heterozygous (n=12) participants. Populations represented by bar graph (G) identifying the average number of cells per cluster divided into HLA-E haplotype and vaccination status.
n = 6
n = 6
HLA-E Heterozygous
n = 12
n = 12
